# New Functional Bionanocomposites by Combining Hybrid Host-Guest Systems with a Fully Biobased Poly(lactic acid)/Poly(butylene succinate-co-adipate) (PLA/PBSA) Binary Blend

**DOI:** 10.3390/jfb14110549

**Published:** 2023-11-15

**Authors:** Francesca Cicogna, Elisa Passaglia, Alice Telleschi, Werner Oberhauser, Maria-Beatrice Coltelli, Luca Panariello, Vito Gigante, Serena Coiai

**Affiliations:** 1 National Research Council-Institute for the Chemistry of OrganoMetallic Compounds (CNR-ICCOM), SS Pisa, Via Moruzzi 1, 56124 Pisa, Italy; elisa.passaglia@pi.iccom.cnr.it (E.P.); alitelleschi@gmail.com (A.T.); 2 National Research Council-Institute for the Chemistry of OrganoMetallic Compounds (CNR-ICCOM), Via Madonna del Piano 10, 50019 Sesto Fiorentino, Italy; woberhauser@iccom.cnr.it; 3 Department of Civil and Industrial Engineering, University of Pisa, Largo L. Lazzarino 1, 56122 Pisa, Italy; maria.beatrice.coltelli@unipi.it (M.-B.C.); luca.panariello@ing.unipi.it (L.P.); vito.gigante@dici.unipi.it (V.G.)

**Keywords:** rosmarinic acid, ferulic acid, glycyrrhetinic acid, layered double hydroxides, PLA/PBSA blend, bionanocomposites, controlled migration, mechanical properties

## Abstract

In this study, we have developed innovative polymer nanocomposites by integrating magnesium-aluminum layered double hydroxide (LDH)-based nanocarriers modified with functional molecules into a fully biobased poly(lactic acid)/poly(butylene succinate-co-adipate) (PLA/PBSA) matrix. These LDH-based hybrid host-guest systems contain bioactive compounds like rosmarinic acid, ferulic acid, and glycyrrhetinic acid, known for their antioxidant, antimicrobial, and anti-inflammatory properties. The bioactive molecules can be gradually released from the nanocarriers over time, allowing for sustained and controlled delivery in various applications, such as active packaging or cosmetics. The morphological analysis of the polymer composites, prepared using a discontinuous mechanical mixer, revealed the presence of macroaggregates and nano-lamellae at the polymer interface. This resulted in an enhanced water vapor permeability compared to the original blend. Furthermore, the migration kinetics of active molecules from the thin films confirmed a controlled release mechanism based on their immobilization within the lamellar system. Scaling-up experiments evaluated the materials’ morphology and mechanical and thermal properties. Remarkably, stretching deformation and a higher shear rate during the mixing process enhanced the dispersion and distribution of the nanocarriers, as confirmed by the favorable mechanical properties of the materials.

## 1. Introduction

Currently, conventional petroleum-based plastics dominate the food packaging industry due to their essential properties, including gas barrier capability, transparency, sealing performance, chemical resistance, mechanical strength, and ease of processing [1,2]. Nonetheless, conventional plastics pose environmental sustainability challenges due to the consumption of nonrenewable resources for their production and improper disposal [3,4].

Despite extensive recycling efforts, a significant amount of plastic packaging remains in the environment. This situation leads to an estimated annual global economic loss of $80–120 billion and raises concerns that if this trend continues, there could be more plastic than fish in our oceans by 2050 [3]. These data underscore the urgent need to adopt more sustainable materials as to petroleum-based single-use plastics.

While replacing traditional plastics with bioplastics does not offer a complete solution to the issues of resource depletion and the proliferation of plastic waste, the use of bioplastics in sectors with extensive plastic use, such as food packaging, presents several opportunities for enhancing their life cycle compared to their fossil-derived counterparts [5,6]. Bioplastics indeed can be mechanically or chemically recycled [7,8] and composted when recycling is no longer feasible.

An example of a promising bioplastic for replacing fossil-derived polymers is poly(lactic acid) (PLA), a thermoplastic polyester that is both bio-derived and compostable under specific conditions, for instance in industrial composting plants. PLA production is also associated with reducing carbon emissions ranging from 15% to 60% and decreasing energy consumption by 25% to 50% compared to petroleum-based polymers [2]. In addition, among the various bioplastics available, PLA possesses favorable mechanical properties and ease of processing through injection molding, extrusion, and blow molding. PLA has also garnered significant interest in food packaging because, in addition to the properties above, it offers practical barriers against oxygen and water vapor and is approved for direct contact with food [9].

However, PLA exhibits specific characteristics that limit its suitability in this sector. PLA’s inherent brittleness and low elongation at break (less than 10%) render it a less competitive material, especially in flexible packaging. It is essential to improve its toughness to make it an ideal candidate in this sector. Researchers have explored various methods to enhance PLA’s performance, including incorporating plasticizers, fillers, reinforcements, and compatibilizers [10,11]. These additives can improve the material’s processability, crystallinity, and barrier properties.

Physically blending PLA with a ductile polymer represents the most straightforward approach for improving the toughness, granting flexibility, and impact resistance [12]. Among the biodegradable and/or compostable polymers, those exhibiting good ductility and easy compatibility with PLA include poly(butylene succinate) (PBS) [13], poly(butylene succinate-co-adipate) (PBSA) [14], poly(caprolactone) [15], and poly(butylene adipate-co-terephthalate) (PBAT) [16]. Researchers have paid particular attention to PLA/PBS and PLA/PBSA blends among these potential combinations of bio-based blends [17,18,19,20,21]. Incorporating PBS or PBSA into PLA has indeed proven to be an effective strategy for reducing PLA’s inherent brittleness while enhancing its flexibility [13,22].

In particular, PBSA stands out for its remarkable impact resistance and flexibility. Additionally, it exhibits excellent characteristics in terms of workability and thermal and chemical resistance and offers a more significant advantage in terms of eco-efficiency compared to other ductile biopolymers [23,24]. As a result, films composed of PLA/PBSA represent a highly compelling solution as potential substitutes for traditional fossil fuel-derived polymers in packaging applications. They have also been demonstrated to be biocompatible and methodologies to modulate their properties by controlled bio-plasticization have been optimized [21].

Coltelli et al. conducted a study on PLA/PBSA blends by varying the ratio of the two polymers [21]. They observed that as the amount of PBSA increased the material became more fluid and easier to process. Moreover, the biodegradability was improved [25]. Furthermore, it was observed that both elongation at break and impact resistance exhibited a notable enhancement with the increase in PBSA content from 5 to 40 wt.%. These changes were associated with a difference in the morphology of the blends from dispersed to co-continuous, as confirmed by scanning electron microscopy (SEM) analysis. Up to 20 wt.% PBSA, the blends had a dispersed phase within a continuous PLA matrix, indicating poor miscibility, while for the 60/40 composition, the morphology changed with the dispersed PBSA phase forming larger structures throughout the matrix [26].

The PLA/PBSA 60/40 blend demonstrated remarkable ductility and toughness, making it highly promising for applications in the packaging industry with a 100% renewable content by weight [27,28]. Additionally, it has recently been shown that industrial films of this blend exhibit excellent tear resistance properties, essential characteristics for a polymer film in the packaging sector [22]. Furthermore, industrial films made of this blend can be recycled by extrusion or injection molding before being composted, thus extending the life cycle, and storing carbon as long as possible. Furthermore, when this blend has been selected as the matrix for biocomposites reinforced with wheat bran or hazelnut shell powder, suitable for rigid packaging applications, promising results have been achieved regarding their recyclability [21,29]. Consequently, the PLA/PBSA 60/40 blend has emerged as a promising material due to its optimized ductility, biocompatibility, and biodegradability balance.

Enhancing specific functional properties becomes invaluable in using the PLA/PBSA 60/40 blend in active packaging and controlled-release applications for hygienic, protective, and personal care products. This effect can be obtained by incorporating gradual and continuous-release antimicrobial or antioxidant additives into the polymer blend. This strategy significantly elevates product quality, food safety, and shelf life by restraining lipid oxidation and inhibiting the proliferation of microorganisms [4,30].

Currently, the active packaging and the hygienic, protective, and personal care products industry prefers employing natural active agents, such as essential oils or polyphenol-rich extracts, to reduce dependence on synthetic additives [31]. Among the numerous natural substances, ferulic acid and rosmarinic acid have garnered extensive attention for their potential applications as antioxidant and antimicrobial additives for plastics [32,33,34,35]. Incorporating ferulic acid into ethylene vinyl alcohol through extrusion blending resulted in films with remarkable antioxidant and antimicrobial features against Gram-negative and Gram-positive bacteria [36]. In contrast, Ordoñez et al. [37] demonstrated that incorporating ferulic acid into PLA through casting and compression molding did not yield significant antimicrobial activity. They concluded that the low molecular mobility of the active compound within the rigid polymer matrix limited its release by affecting the antimicrobial capacity or that the ferulic acid reacted with the PLA terminal groups. However, Sharma et al. [38] reported that adding ferulic acid to a PLA/PBAT blend by the solvent casting method conferred UV barrier properties and effective antimicrobial activity, thus preventing the growth of pathogenic bacteria like Listeria monocytogenes and Escherichia coli on packaged food products.

The direct incorporation of rosmarinic acid into polymer matrices has been less explored than that of ferulic acid. For example, Kahya et al. [39] showed that chitosan films mixed with sage and rosemary extracts containing rosmarinic acid possess antioxidant properties and increased antimicrobial capacity compared to untreated chitosan. The data also revealed that rosmarinic acid prevents oxidative deterioration of the polymer film and migrates by diffusing into the food.

Another organic acid with interesting properties is 18β-glycyrrhetinic acid, obtained by hydrolysis of glycyrrhizic acid extracted from licorice. This acid is primarily used in pharmacological and cosmetic applications for its proven antimicrobial and anti-inflammatory activity [40,41]. Recently, nanostructured systems based on chitin-nano lignin nanofibrils and encapsulated 18β-glycyrrhetinic acid have been prepared, which, when incorporated into PLA or deposited on the surface of PLA films, have shown cytocompatibility and anti-inflammatory activity [42].

A polymeric material designed for active packaging should exhibit a controlled release of encapsulated active molecules. This controlled release is essential to maintain the efficacy of these molecules throughout the product’s shelf life. Various strategies can be employed to achieve this objective, including immobilizing active molecules within organic or inorganic systems, which are dispersed within the polymer matrices or deposited on their surfaces [43,44].

Among the various methods described in the literature, the use of Layered Double Hydroxides (LDHs) to create functional hybrid systems suitable for active packaging emerges as one of the most promising. The unique structure of LDHs, composed of nanometrically thick mixed hydroxide layers that are positively charged and separated by anions, allows for the intercalation or adsorption of active molecules of different chemical nature, ensuring an effective balance between the number of released molecules and the time required to exert their activity in a packaging application [45,46,47]. This approach is particularly advantageous for substances sensitive to light, oxygen, and temperature, such as phenolic acids, as it also aims to preserve their biological activity [48].

Numerous examples of preparation, characterization, and application of LDH-based hybrid systems modified with organic and synthetic acids with antioxidant and antimicrobial activity are reported in the literature, such as salicylic acid, glycolic acid, citric acid, and aleuritic acid [49]. LDHs modified with these molecules have been effectively dispersed in poly(ethylene terephthalate) and low-density polyethylene (LDPE), demonstrating the transfer of antimicrobial activity to the polymeric matrix and the ability for controlled release of the molecule over time [50].

Our research group previously synthesized LDH modified with rosmarinic acid (LDH-RA), effectively dispersed in LDPE and PLA through melt mixing [47,51]. The collected data demonstrated that the composite materials inhibit the growth of Staphylococcus aureus and, for PLA, also of Escherichia coli. Moreover, preliminary studies have highlighted a controlled release of the active compound from composite film samples immersed in a 95% ethanol solution. Additionally, a significant stabilizing effect on the thermal oxidative degradation of LDPE has been observed due to the antioxidant properties of rosmarinic acid. LDH-RA and LDH modified with 18β-glycyrrhetinic acid have also been added to polyester blends using the solution casting method, demonstrating a similar effect in controlling the migration of active molecules. However, as industrial applications rely on material transformation through hot-melt extrusion compounding methods, it is necessary to verify the characteristics of the composite material obtained by melt mixing.

**Figure 1:**
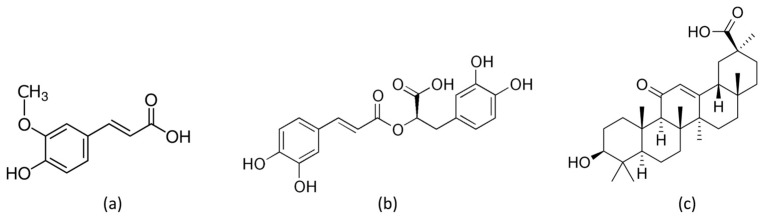
Molecular structure of (**a**) trans-ferulic acid (FA-H), (**b**) rosmarinic acid (RA-H), and (**c**) 18β-glycyrrhetinic acid (GA-H).

In this study, we have prepared magnesium-aluminum LDH-based lamellar nanoparticles modified with mono-deprotonated anions derived from ferulic acid (FA-H), rosmarinic acid (RA-H), and 18β-glycyrrhetinic acid (GA-H) (Figure 1). We have evaluated these nanoparticles as additives in the PLA/PBSA 60/40 blend to produce bio-nanocomposites with functional properties for the active packaging sector. In contrast to previous research, we have prepared the composites using melt-processing methods. We have conducted a comparative analysis between a batch-processing approach and a continuous process conducted at a laboratory scale. This study delves into the structural, thermal, and morphological properties of the composites produced through both processing methods. Additionally, we have assessed water vapor permeability and investigated the release kinetics of active molecules within a food simulant.

## 2. Materials and Methods

### 2.1. Materials

The magnesium-aluminum layered double hydroxide intercalated with nitrate anion (LDH-NO_3_) with the molecular formula [Mg_0.66_Al_0.34_(OH)_2_](NO_3_)_0.34_·0.5H_2_O was purchased from Prolabin&Tefarm (Perugia, Italy). The reagents used for the modification of LDH-NO_3_ are as follows: trans-ferulic acid (FA-H) 99% (C_10_H_10_O_4_, MW: 194.18 g/mol, CAS number 537-98-4), rosmarinic acid (RA-H) 96% (C_18_H_16_O_8_, MW: 360.31 g/mol, CAS number 20283-92-5), and 18β-glycyrrhetinic acid (GA-H) 97% (C_30_H_46_O_4_, MW: 470.7 g/mol, CAS number 471-53-4), all purchased from Sigma-Aldrich (Milan, Italy) and used as received without further purification. 2,2-diphenyl-1-picrylhydrazyl (DPPH) (C_18_H_12_N_5_O_6_, MW: 394.33 g/mol, CAS number 1898-66-4) and 6-hydroxy-2,5,7,8-tetramethylchroman-2-carboxylic acid (Trolox) 97% (C_14_H_18_O_2_, MW: 250.3 g/mol, CAS number 53188-07-1), both purchased from Sigma-Aldrich, were used as received.

For the polymer matrix, we used a 60/40 weight mixture of PLA/PBSA. PLA IngeoTM 2003D, which is composed of 4.1% D isomer and has a density of 1.24 g/cm^3^, was produced by NatureWorks LLC (Minnetonka, MN, USA). PBSA bioPBS FD92PM, which is a copolymer of succinic acid, adipic acid, and 1,4-butanediol and has a density of 1.24 g/cm^3^, was produced by Mitsubishi Chemical Corporation (Tokyo, Japan). The solvents used as received are as follows: ACS reagent grade chloroform ≥ 99.8% and HPLC grade chloroform ≥ 99.8%, stabilized with ethanol, from Sigma-Aldrich (St. Louis, MO, USA) (CHCl_3_, MW: 119.38 g/mol, CAS number 67-66-3); ≥99.5% extra pure methanol from Acros Organics (Geel, Belgium) (CH_3_OH, MW: 32.04 g/mol, CAS number 67-56-1); ACS reagent grade ethanol 96% from Sigma-Aldrich (C_2_H_5_OH, MW: 46.06 g/mol, CAS number 64-17-5); ACS grade tetrahydrofuran (THF) ≥ 99.5%, stabilized with BHT, from Sigma-Aldrich (C_4_H_8_O, MW: 72.11 g/mol, CAS number 109-99-9).

All suspensions were prepared using ultrapure water (18.2 Mohm·cm) obtained with a Milli-Q system (Millipore, Bedford, MA, USA).

### 2.2. Preparation of Modified LDHs (LDH-FA, LDH-RA, and LDH-GA)

LDH-FA (ferulic acid-modified LDH), LDH-RA (rosmarinic acid-modified LDH), and LDH-GA (glycyrrhetinic acid-modified LDH) were synthesized following a previously reported method [47,52]. The anion exchange reactions were conducted using CO_2_-free water. For LDH-FA preparation, 1.47 g (7.57 mmol) of FA-H, corresponding to 1 time the anion exchange capacity (AEC) of LDH-NO_3_, equivalent to 3.80 meq/g, were added to 100 mL of water. Subsequently, 398 µL of 50% (*w*/*w*) NaOH was added under a nitrogen atmosphere, followed by 2 g of LDH-NO_3_. The suspension was stirred for 24 h at room temperature. The resulting LDH-FA product was recovered through centrifugation, washed with water, and dried under vacuum at 40 °C for 24 h until a constant weight was attained (2.44 g). From the UV-vis analysis, it was determined that the content of FA in LDH-FA is 37 wt.%.

To obtain LDH-RA, 2.74 g (7.60 mmol) of RA-H, corresponding to 1 time the AEC of LDH-NO_3_, were dissolved in 100 mL of water, and 10 mL of ethanol was added to aid in RA-H solubilization. Under a nitrogen atmosphere, LDH-NO_3_ (2 g) was added, and the suspension was stirred for 48 h at room temperature. The LDH-RA product was recovered through centrifugation, washed with water, and dried under vacuum at 40 °C for 24 h until a constant weight was achieved (2.48 g). From the UV-vis analysis, it was determined that the content of RA in LDH-RA is 46 wt.%.

Finally, for LDH-GA synthesis, the monosodium salt of GA-H (Na-GA) was first prepared using the method reported by Wu et al. [53]. Subsequently, 1.79 g (3.64 mmol) of Na-GA, corresponding to 1 time the AEC of LDH-NO_3_, were dissolved in 50 mL of water/ethanol 60/40 (*v*/*v*). Under a nitrogen atmosphere, LDH-NO_3_ (1 g) was added, and the suspension was stirred for 24 h at room temperature. The LDH-GA product was recovered through centrifugation, washed with deionized water, and dried under vacuum at 40 °C for 24 h until a constant weight was achieved (0.85 g). From the UV-vis analysis, it was determined that the content of GA in LDH-GA is 17 wt.%.

### 2.3. Preparation of Polymer Nanocomposites

#### 2.3.1. Melt Compounding in a Batch Mixer

The composites were prepared by melt blending in a Brabender OHG47055 mechanical mixer (Belotti, Milan, Italy). For each type of modified LDH, two master-batches were previously prepared in solution by mixing the modified LDH with PBSA. The master-batches were then diluted in the mechanical mixer with a PLA and PBSA mixture, so that the final composites contained 1 wt.% or 4 wt.% of modified LDH relative to the polymer matrix, and the matrix composition was 60/40 *w*/*w* PLA/PBSA. The melt compounding was carried out at 180 °C, with a rotation speed of 50 rpm, and for a processing time of 10 min. A pure PLA/PBSA blend was also prepared. Before melt mixing, PLA and PBSA were vacuum dried at 80 °C for 18h.

The following procedure was used for the preparation of the master-batches. The modified LDH was suspended in chloroform, and the suspension was sonicated for 10 min with a Hielscher Ultrasonic Processor UP200St (200 W, 26 kHz, Hielscher Ultrasonics, Teltow, Germany) equipped with a titanium 2 mm sonotrode (S26d2). At the same time, PBSA was dissolved in chloroform, and the LDH suspension was added drop by drop to this solution (2 g of PBSA in 30 mL of CHCl_3_) and kept under stirring for 2 h at room temperature. The sample was recovered by evaporating the solvent under a vacuum and finally dried under a vacuum at room temperature for 24 h.

The weight percentage of FA incorporated in LDH-FA is 37%. Consequently, the weight percentage of FA in the PLA/PBSA/LDH-FA_1 and PLA/PBSA/LDH-FA_4 nanocomposites is 0.37% and 1.48%, respectively. When LDH-RA is employed, with an RA content of 46 wt.% in the hybrid system, the weight percentage of RA in the PLA/PBSA/LDH-RA_1 and PLA/PBSA/LDH-RA_4 nanocomposites is 0.46% and 1.84%, respectively. Finally, with an amount of GA in LDH-GA comprising 17 wt.%, the weight percentage of GA in the PLA/PBSA/LDH-GA_1 and PLA/PBSA/LDH-GA_4 nanocomposites are 0.17% and 0.68%, respectively.

All nanocomposites were filmed and subsequently characterized. Films of approximately 100 μm in thickness were obtained by compression molding with a Carver 3851-0 press (Carver, Inc. Wabash, IN, USA), setting the plate temperature to 180 °C and applying a pressure of 4 bar for 1 min.

#### 2.3.2. Melt Compounding in a Micro-Compounder

PLA/PBSA/LDH nanocomposites were prepared in a micro-compounder Haake Minilab II (Thermo Scientific Haake GmbH, Karlsruhe, Germany), which consists of two co-rotating conic screws. This micro-compounder combines both melt processing operations and measurements of torque.

For each extrusion trial, 3.6 g of PLA pellets and 2.4 g PBSA pellets were weighted in a glass becker and the LDH-RA (1 wt.%) in powder was added and manually mixed. The pellets were previously dried in an oven at 60 °C for 16 h. Once the desired temperature was reached, the calibration of pressure transducers, before the beginning of measurements, was conducted. The mixture was fed into the co-rotating micro-compounder manually through a little hopper. After the introduction of the material, the melt, pushed by the screws, runs through a closed circuit (with the valve closed) for 1 min, during which the torque is measured as a function of time. The material was recovered after one minute of flow inside the chamber to ensure correct mixing. Acceptable values were obtained from at least five experimental tests to guarantee the reliability and consistency of the test and to recover enough material for further tests. In the experiments, the rotating speed was 100 rpm and 150 rpm, and the processing temperature was 190 °C. The final torque value represents the most significant value for the sample as the melt stabilizes. With the opening of the valve, the melted filament was collected by a heated cylinder piston and fed into a mini-injection molding machine (Thermo Scientific HAAKE Minijet II, Karlsruhe, Germany), to produce specimens for the tensile tests (25 × 5 × 1 mm). The mold was held at 55 °C for an injection cycle of 5 s, 540 bar of injection pressure and 200 bar of post pressure were set.

### 2.4. Characterization

X-ray diffraction (XRD) analysis was conducted at room temperature using an X’PERT PRO (PANalytical, Malvern, UK) powder diffractometer equipped with Cu Kα radiation (wavelength of 1.541874 Å), a nickel filter with a thickness of 0.02 nm, and a fast detector (PIXcel) with an active range of 3.347°. Spectra were acquired in the 2θ range from 1.5 to 30°, employing a step size of 0.0131° and a counting time of 207.5 s. The basal distance of the LDH was determined from the diffraction signal (003) using Bragg’s law. The modified LDH samples were characterized as powders, while the polymer composites were analyzed as films. Prior to analysis, the polymer samples underwent annealing in a vacuum oven at 80 °C for 6 h.

Infrared spectra (FT-IR) were collected with a Fourier Transform Spectrometer (Spectrum Two, PerkinElmer, Waltham, MA, USA) in the range of 400–4000 cm^−1^ with a resolution of 4 cm^−1^, averaging 16 scans. The spectra of LDHs and organic anions were obtained by mixing the samples with potassium bromide. Data processing was performed using the Spectrum software (version 10.4.2, 2014, PerkinElmer, Waltham, MA, USA) provided by the instrument.

UV-vis absorption spectra were collected at room temperature using a Jasco V-750 UV-visible spectrophotometer (Jasco International Co., Ltd., Tokyo, Japan). Calibration curves of FA-H, Na-FA, RA-H, GA-H, and Na-GA were obtained by measuring the absorbance of solutions at different concentrations and fitting the data as a function of the molar concentration. The absorbance was measured at the maximum wavelength for each molecule, which was 323 nm for FA-H, 311 nm for Na-FA, 333 nm for RA-H, 250 nm for GA-H, and 250 nm for Na-GA. The molar extinction coefficients were calculated from the linear fitting of each analysis: FA-H ε_323_ = 15,700, Na-FA ε_311_ = 14,600, RA-H ε_333_ = 18,800, GA-H ε_250_ = 10,700 M^−1^ cm^−1^, and Na-GA ε_250_ = 10,400 M^−1^ cm^−1^. The quantification of organic anions (FA and RA) in the corresponding LDHs was performed by dissolving a known amount (approximately 5 mg) of each modified LDH in 25 mL of a 1M HCl aqueous solution [54]. The determination of the GA content was carried out by dissolving a known amount (2–3 mg) of the corresponding modified LDH in a few drops of concentrated HCl, followed by dilution in EtOH [47]. After appropriate dilution, UV-vis spectra of the solutions were recorded, and the amount of organic fraction present in the modified LDHs was determined.

Thermogravimetric analysis (TGA) was performed using an SII TG/DTA 7200 EXSTAR instrument from Seiko (Chiba, Japan). For LDH samples, 5–10 mg of materials were placed in 70 μL alumina sample pans and analyzed under an air flow of 200 mL min^−1^ at a heating rate of 10 °C min^−1^ from 30 to 900 °C. For PLA/PBSA nanocomposites, 5–10 mg of the samples were placed in 70 μL alumina sample pans and analyzed under a nitrogen flow of 200 mL min^−1^ at a heating rate of 10 °C min^−1^ from 30 to 700 °C.

Size exclusion chromatography (SEC) analysis was performed using an Agilent Technologies 1260 Series instrument (Agilent, Santa Clara, CA, USA) equipped with a degasser, an isocratic HPLC pump, a refractive index (RI) detector, a PLgel 5 μm pre-column, and two PLgel MiniMIX-D 5 μm columns (Agilent, Santa Clara, CA, USA) conditioned at 35 °C. CHCl_3_ for HPLC was used as the eluent at a flow rate of 0.3 mL min^−1^. The system was calibrated with polystyrene standards in a range from 500 to 3 × 10^5^ g mol^−1^. The samples were dissolved in CHCl_3_ (3 mg mL^−1^) and filtered through a 0.20 μm syringe filter before analysis. The number average molecular weight (M_n_) and weight average molecular weight (M_w_) were calculated using Agilent ChemStation software (OpenLab Control Panel version A0104, Agilent Technologies, Santa Clara, CA, USA). The PLA fraction was separated from the PBSA fraction, and both fractions were analyzed separately. The separation of the two polymer phases was conducted by suspending the composite in THF, which dissolved the PLA but not the PBSA. The two polymers were then recovered by filtration.

The morphological analysis was performed using a FEI Quanta 450 FEG scanning electron microscope (SEM) (Thermo Fisher Scientific, Waltham, MA, USA, instrument located at CISUP—Center for Instrumentation Sharing—University of Pisa). The modified LDH samples were characterized as powders, while the polymeric nanocomposites were characterized as films, which were cryo-fractured in liquid nitrogen. The cryo-fractured surface was covered with a tiny metallic layer of Pt, in a way that the surface could be electrically conductive.

The water vapor permeability tests were conducted using an XS/Pro-PermeH2O ExtraSolution instrument (ExtraSolution, Lucca, Italy). The samples were analyzed as films with a thickness of approximately 170–190 µm and a surface area of 50 cm^2^. Prior to analysis, the samples were conditioned for 8 h inside the instrument, exposing one side to 50% relative humidity and the other side kept dry. The temperature was set at 23 °C during the tests. Dry nitrogen gas with a flow rate of 11.80 mL min^−1^ was used as the carrier gas, and the concentration of water vapor within the nitrogen flow was measured by an integrated infrared detector. The analysis automatically stopped when the Water Vapor Transmission Rate (WVTR) reached a constant value, with signal variations below 0.5% compared to the mean value. To facilitate comparison among different samples, the WVTR values obtained from each film analysis were normalized to a uniform thickness of 180 µm.

Tensile tests were performed on HAAKE Type 3 specimens (HAAKE, Vreden, Germany) (width = 5 mm, thickness 1.5 mm, and useful length of 25 mm) injection molded by the MiniJet press (Thermo Scientific HAAKE Minijet II, Karlsruhe, Germany). The machine was equipped with a 1 kN load cell, manual grips, and interfaced with MERLIN software (INSTRON version 4.42 S/N–014733H, INSTRON, Canton, MA, USA). The initial grip separation was 25 mm, and the deformation rate was set at 10 mm/min. For the evaluation of elastic modulus an analogic extensometer has been used. Statistical analysis was carried out to discuss the significance of the differences observed between the obtained values of stiffness, mechanical resistance, and ductility setting a significance threshold of 0.05. *p*-values obtained from the statistical analysis were reported for each considered sample. A *p*-value less than 0.05 indicates that there is less than 5% probability that the results are not significantly different.

### 2.5. DPPH Test

The antioxidant activity of FA-H, RA-H, LDH-FA, and LDH-RA was determined using the DPPH method. Methanol solutions of DPPH (6 × 10^−5^ M), RA-H (1.6 × 10^−4^ M), and Trolox (2 × 10^−3^ M) were prepared. Suspensions of LDH-RA and LDH-FA (2 mg in 4 mL) were also prepared and sonicated for 10 min to promote delamination of organophilic LDH. Three mL of the DPPH solution were mixed with different aliquots of FA-H or RA-H solutions to obtain final concentrations of FA-H ranging from 2.9 × 10^−5^ M to 7.3 × 10^−6^ M, and RA-H ranging from 4.18 × 10^−6^ M to 1.05 × 10^−6^ M. MeOH was added to each solution to reach a final volume of 3.08 mL. Additionally, a blank DPPH solution was prepared by adding MeOH to the DPPH solution. In the case of LDH-FA and LDH-RA, 3 mL of the DPPH solution were mixed with different aliquots of LDH-FA or LDH-RA suspension and MeOH was also added to reach a final volume of 3.18 mL. Considering the organic fraction present in each modified LDH, the final FA concentration in the DPPH solutions ranged from 5.6 × 10^−5^ M to 3.1 × 10^−6^ M, and the final RA concentration ranged from 3.8 × 10^−5^ M to 2.1 × 10^−6^ M. For the Trolox analysis, the final concentrations in the DPPH solutions ranged from 6.5 × 10^−5^ to 1.3 × 10^−5^ M.

All solutions were kept in the dark for 24 h, and their UV-vis spectra were recorded, with the absorbance at 515 nm measured. Each sample (FA-H, LDH-FA, RA-H, LDH-RA) was analyzed three times, and the average values of the parameters were reported. The percentage of DPPH reduction (I%) (Equation (1)) was calculated as a function of the antioxidant concentration, and linear fitting of the experimental data was performed. The EC_50_ value was determined as the antioxidant concentration corresponding to I% = 50%.

(1) $$I\%=\frac{\left(A_{0}-A_{t}\right)}{A_{0}}\times 100$$

where: A_0_ is the absorbance of the DPPH solution in the absence of an antioxidant; A_t_ is the absorbance of the DPPH solution in the presence of an antioxidant at the end of the reaction.

The DPPH test was also conducted on a polymeric film of the PLA/PBSA_LDH-RA_4 sample by suspending approximately 27.3 mg of the film (thickness approximately 150 μm) in 3 mL of a DPPH solution (6 × 10^−5^ M) and monitoring the disappearance of the peak at 515 nm as a function of time. The same film was then suspended in 3 mL of a freshly prepared DPPH solution, and the disappearance kinetics of the peak at 515 nm were monitored again.

### 2.6. Migration Test

Migration tests were carried out on PLA/PBSA/LDH composites containing 4 wt.% of modified LDH. Approximately 100 mg of each film, 150 µm thick, was placed in contact with 25 mL of a 50/50 (*v*/*v*) EtOH/H_2_O solution at room temperature, and continuous stirring at 250 rpm was maintained for approximately 30 consecutive days. The release kinetics of the anions (FA, RA, GA) from the polymer matrix were monitored using UV-vis spectroscopy by recording spectra of the extracting solution at regular time intervals. For comparison, samples of PLA/PBSA containing the pure organic acid, FA-H, RA-H, and GA-H, in the same amount present in the 4 wt.% PLA/PBSA_LDH composites, were prepared by solution mixing and characterized as films.

Migration tests were also carried out on films of PLA/PBSA/LDH-RA_1 prepared in the batch mixer and in the micro-compounder 100 and 150 rpm by applying the same procedure described previously.

To model the release kinetics, we employed the Peleg equation (Equation (2)): this allowed us to calculate the equilibrium mass of the active compound (M_∞_), the partition coefficient (M_∞_/M_0_), and the release rate [55].

(2) $$\frac{t}{M_{t}}=k_{1}+k_{2}t$$

where: M_t_ is the mass of active compound released at time t; k_1_ and k_2_ are the constants of the model; k_1_ is inversely proportional to the initial release rate (1/k_1_), and k_2_ is related to the asymptotic release value at equilibrium (M_∞_ = 1/k_2_).

Furthermore, experimental data were also analyzed using the Korsmeyer–Peppas model or power-law (Equation (3)), which is valid for up to 60% of the total release of the active molecule [56]:

(3) $$\frac{M_{t}}{M_{\infty }}=kt^{n}$$

where: M_t_/M_∞_ is the fraction released at time t relative to the equilibrium concentration (as t approaches infinity); k is the release rate constant that incorporates various factors involved in the diffusion process, including the macromolecular characteristics of the film and the molecular properties of the active compound; n is a constant indicating the release mechanism. Particularly, for a thin film sample, a value of n equal to 0.5 indicates Fickian diffusion, values of n less than 0.5 are considered nearly Fickian diffusion, while values of n greater than 0.5 are known as anomalous transport, involving both polymer relaxation and diffusion of the active compound within the polymer. Data fitting with the two models was performed using Origin 2016 software (OriginLab Corporation, Northampton, MA, USA).

## 3. Results

### 3.1. Characterization of Functional LDHs

**Figure 2:**
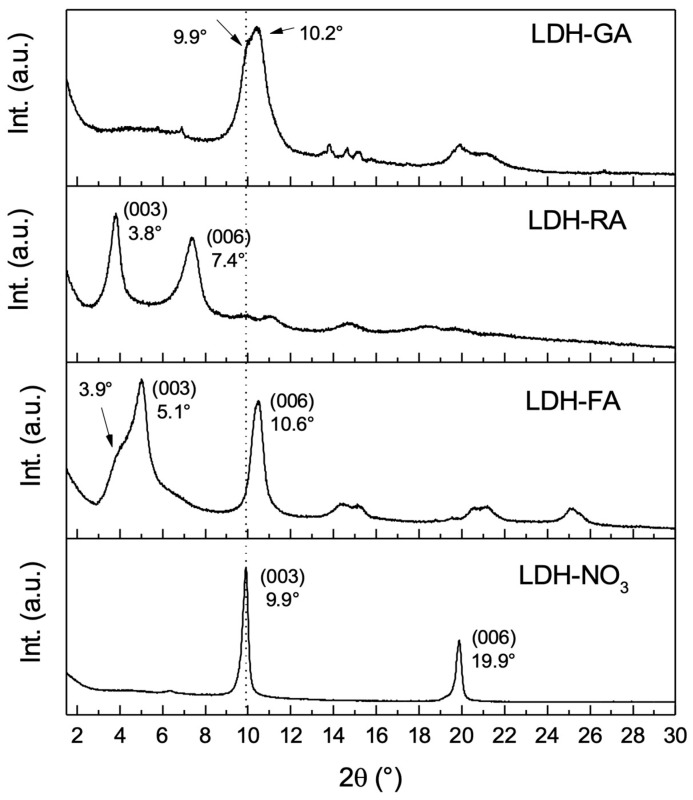
XRD patterns of LDH-NO_3_, LDH-FA, LDH-RA, and LDH-GA.

Three functional LDHs were synthesized by starting from LDH-NO_3_ and replacing nitrate anions with carboxylate anions derived from FA-H, RA-H, and GA-H compounds. For all three host-guest nanosystems, FT-IR analysis showed the presence of vibration signals characteristic of both the inorganic and organic constituents (Supplementary Material Figure S1), thus confirming the successful immobilization. Nonetheless, this analysis did not conclusively discern whether the anions were intercalated within the layers or merely adsorbed onto them. To delve into this aspect, the samples underwent characterization using XRD analysis to probe the potential expansion of the interlayer spacing caused by the insertion of organic molecules between the layers (Figure 2).

The XRD diffractogram of LDH-FA revealed noteworthy changes in the diffraction peaks associated with (003) and (006) reflections, previously observed in LDH-NO_3_ at angles of 9.9° and 19.9°, respectively. In the case of LDH-FA, these peaks shifted to 5.1° and 10.6°, indicative of an expansion in the basal spacing from 0.9 nm to 1.7 nm. An interesting aspect emerged with the (003) reflection, where an additional signal, partially overlapping around 3.9°, appeared. This phenomenon aligns with existing literature findings, suggesting the arrangement of ferulate anions in a bilayered configuration rather than a monolayered one [57].

The XRD diffractogram of LDH-RA showed a distinctive pattern. The (003) reflection emerged at 3.8° and the (006) reflection at 7.6°. This shift indicates an increase in the basal distance from 0.9 nm to 2.3 nm.

However, the XRD pattern of LDH-GA diverged from the observed trends. Unlike LDH-FA and LDH-RA, the (003) reflection associated with LDH-NO_3_ did not shift to lower 2θ angles. Instead, a splitting of the signal occurred, with one peak aligning closely with LDH-NO_3_’s (003) reflection at approximately 9.9°, and another peak around 10.2°. This divergence suggests a contraction in the basal spacing, rather than an expansion. Considering the dimensions of the GA anion [47], this outcome implies that intercalation between the layers had not been achieved. Nevertheless, the confirmation of the hybrid nature of the product through FT-IR analysis provides reasonable grounds to infer that the GA anion has been adsorbed. Furthermore, the discernible shift towards lower diffraction angles in LDH-GA’s XRD profile could feasibly signify that during the modification or washing steps of LDH-GA synthesis, some nitrate anions from the LDH-NO_3_ precursor may have undergone partial substitution by carbonate anions [47].

**Figure 3:**
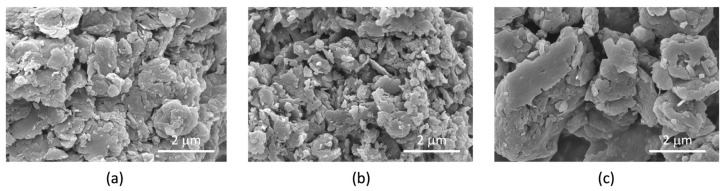
SEM micrographs of LDH-FA (**a**), LDH-RA (**b**), and LDH-GA (**c**).

The SEM micrographs of LDH-FA and LDH-RA revealed an irregular platelet-like morphology, exhibiting average lateral dimensions spanning from a few hundred nanometers to 1–2 µm, accompanied by noticeable particle aggregation (Figure 3). In the case of LDH-GA, the aggregates appeared larger compared to LDH-FA and LDH-RA and were characterized by a smoother surface, likely due to the adsorption of GA and the consequent surface coating of the particles. Because of the organophilic coating, it is indeed probable that hydrophobic interactions promoted greater particle connectivity, culminating in the formation of larger agglomerates [58].

The quantification of organic components within the hybrid systems was conducted by acquiring UV-vis spectra of solutions derived from solubilizing the LDH in an acidic environment, following the methodology outlined in the experimental section. The resulting spectra displayed distinct absorption peaks corresponding to the respective organic acidic species: at λ = 323 nm for LDH-FA, λ = 333 nm for LDH-RA, and λ = 250 nm for LDH-GA. Analysis of the data revealed that LDH-FA incorporated 37 wt.% of the ferulate anion, which was effectively immobilized within the lamellar structure. In the case of LDH-RA, the rosmarinate anion was found to constitute 46 wt.%, while LDH-GA exhibited the glycyrrhetinate anion at 17 wt.%.

The thermal degradation characteristics of the hybrid systems were investigated using TGA analysis. Indeed, the thermograms play a crucial role in confirming the presence of the organic fraction and provide insight into the starting temperature of the degradation process related to the immobilized organic content.

**Figure 4:**
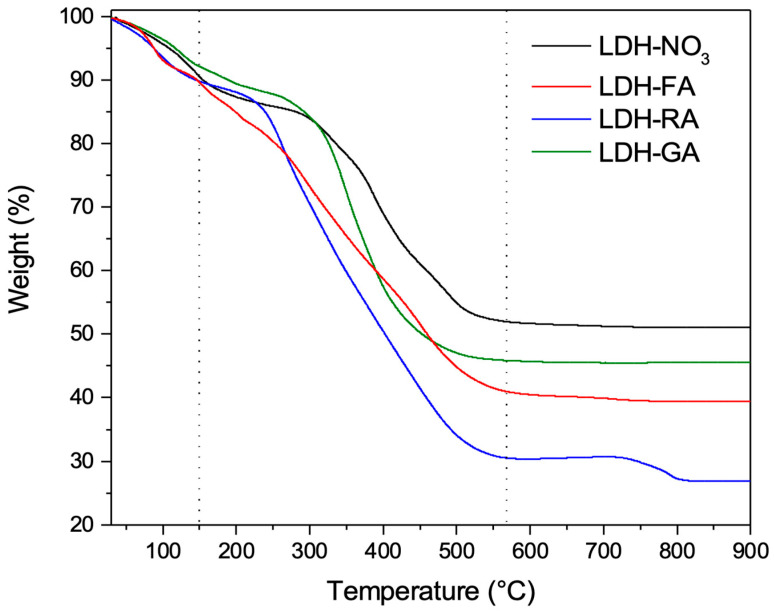
TGA thermograms of LDH-NO_3_, LDH-FA, LDH-RA, and LDH-GA.

In the thermograms of the functional LDHs (Figure 4), an initial weight loss was clearly observed, corresponding to the evaporation of adsorbed or intercalated water [58]. Subsequently, a complex degradation pattern unfolded, wherein the thermal degradation of the organic component occurred in successive steps, depending on the complexity of the involved anion. Alongside this, layer dehydroxylation and the degradation of residual inorganic anions took place, leading to the formation of mixed magnesium and aluminum oxides as the ultimate residue.

Upon a detailed analysis of the thermograms, a noticeable pattern emerged: the onset of degradation for LDH-FA, excluding the initial water evaporation phase, was the lowest among the three systems, occurring at approximately 150–180 °C. In contrast, this threshold was higher, at 200–250 °C, for LDH-RA and surpassed 250 °C for LDH-GA. The relatively modest thermal stability of LDH-FA can be attributed to the desorption and decomposition of ferulate anions, processes that take place at lower temperatures compared to the bulkier and more robust RA and GA compounds [59].

### 3.2. PLA/PBSA/LDH Nanocomposites via Batch Mixing

#### 3.2.1. Structure and Morphology

During the preparation of the PLA/PBSA/LDH nanocomposites using a batch mixer, the torque trend was monitored. It was noted that the torque values closely resembled those of the PLA/PBSA mixture, except for one significant variation: the inclusion of LDH-GA resulted in a reduction in the torque value (Figure S2). This reduction could potentially arise from a decrease in the molecular weight of the polymer matrix, or a plasticizing effect exerted by LDH-GA. These possibilities will be subjected to further investigation in the subsequent sections.

**Figure 5:**
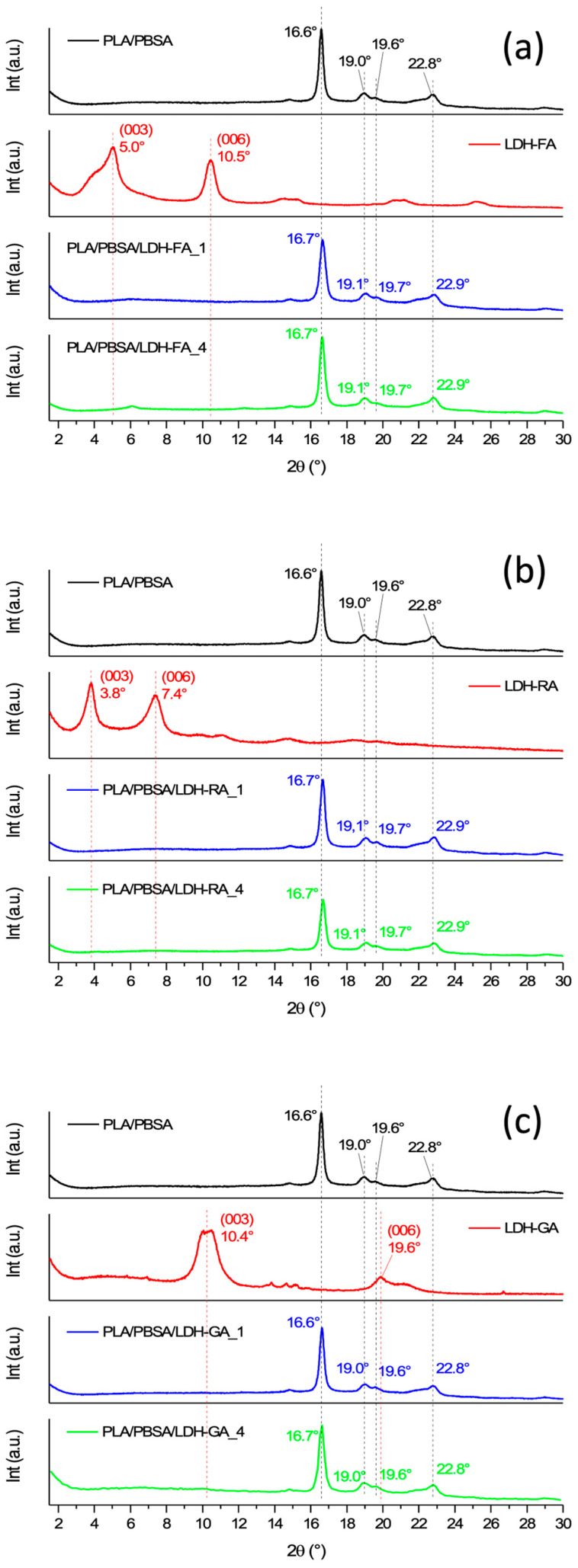
XRD patterns of LDH-FA, PLA/PBSA, PLA/PBSA/LDH-FA_1, PLA/PBSA/LDH-FA_4 (**a**), LDH-RA, PLA/PBSA, PLA/PBSA/LDH-RA_1, PLA/PBSA/LDH-RA_4 (**b**), LDH-GA, PLA/PBSA, PLA/PBSA/LDH-GA_1, PLA/PBSA/LDH-GA_4 (**c**).

The dispersion of functional LDH within the polymer matrix was evaluated through XRD analysis. All collected diffraction patterns showed distinct reflections from the crystalline segment of PLA at 16.6°, 19.0°, and 22.3°, corresponding to the (110)/(200), (203)/(113), and (211) diffraction planes, respectively [60,61]. Similarly, the alpha crystalline fraction of PBSA was discernible at 19.6°, 21.9°, and 22.8°, aligned with the diffraction planes (020), (021), and (110) [62] (Figure 5).

No low-angle diffraction signals were observed in composite materials containing 1 wt.% LDH. This evidence implies the absence of undispersed LDH, indicating the potential formation of an exfoliated morphology. Conversely, in composites containing 4 wt.% LDH, faint signals emerged at lower angles, consistent with (003) LDH diffraction. Surprisingly, the XRD diffraction pattern of the PLA/PBSA/LDH-FA_4 sample displayed a weak signal around 6° corresponding to a narrower interlamellar spacing than that of LDH-FA. This result suggests that a contraction of the interlamellar space of the dispersed LDH-FA within the polymer matrix likely occurred during mixing.

To delve deeper into the morphological differences among the various samples, both the pure blend and the composites underwent SEM characterization. The PLA/PBSA blend showed a co-continuous structure characterized by the presence of discrete phases in the form of polymer droplets, indicating limited miscibility (Figure S3). Nevertheless, the presence of filaments suggests a ductile behavior of the blend, even at cryogenic fracture temperatures.

**Figure 6:**
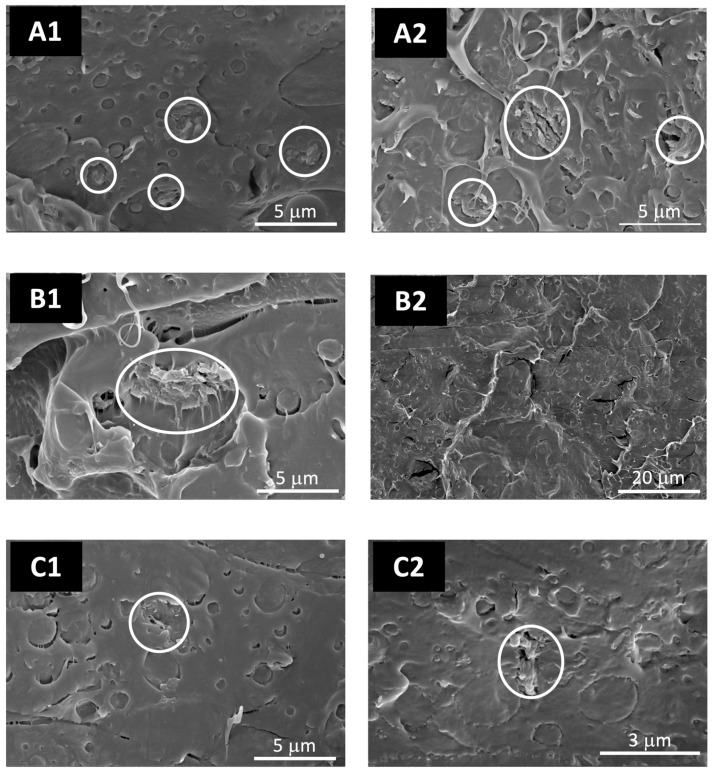
SEM micrographs (ETD detector) of PLA/PBSA/LDH-FA_1, PLA/PBSA/LDH-RA_1, and PLA/PBSA/LDH-GA_1 (**A1**,**B1**,**C1**), and of PLA/PBSA/LDH-FA_4, PLA/PBSA/LDH-RA_4, and PLA/PBSA/LDH-GA_4 (**A2**,**B2**,**C2**) at different magnifications. Microaggregates of LDH particles are indicated by the white circles.

**Figure 7:**
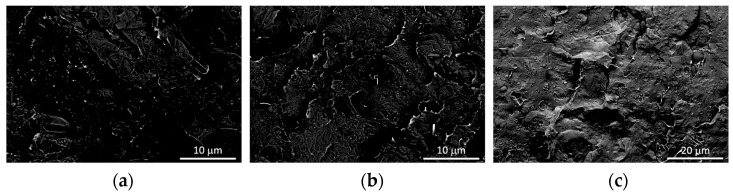
SEM micrographs (CBS detector) of PLA/PBSA/LDH-FA_4 (**a**), PLA/PBSA/LDH-RA_4 (**b**), and PLA/PBSA/LDH-GA_4 (**c**).

In composite materials, the morphology of the polymer matrix closely resembled that of the blend. Nevertheless, the separation between the two polymeric phases in the composites becomes less distinct than in the pure blend, suggesting the enhanced compatibility between PLA and PBSA (Figure 6). Regarding the dispersion of LDHs, SEM micrographs revealed the presence of micro-aggregates in all composite samples. These aggregates, marked by white circles in Figure 6, can be attributed to the diverse LDH types. However, micrographs obtained from samples containing 4 wt.% of the LDH, utilizing the backscattered electron detector, revealed the presence of numerous submicron-sized particles that are uniformly dispersed within the polymer matrix (Figure 7). In these images, the prevalence of intense focal points, corresponding to LDH, contrasts against the polymer matrix’s dark backdrop, confirming the LDH’s presence in the composites.

**Figure 8:**
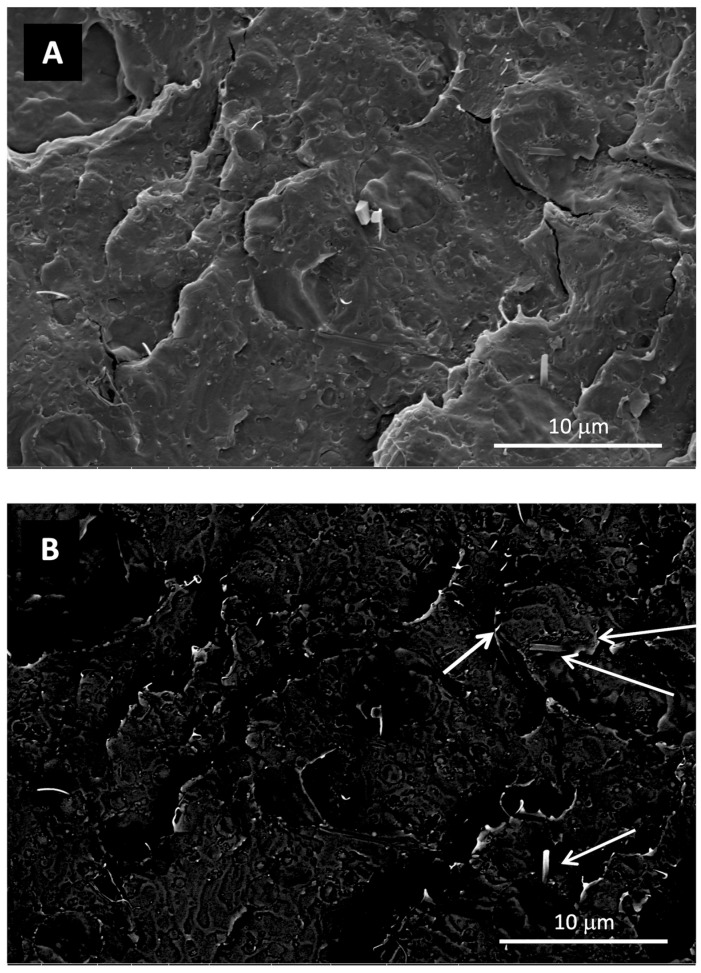
Comparison between SEM micrographs of the PLA/PBSA/LDH-GA_4 sample acquired at the same magnification and position using both secondary electrons (**A**) and backscattered electrons (**B**). The LDH nanoparticles are indicated by the white arrows.

Compared to the other samples, improved dispersion of LDH particles was evident in the composites containing LDH-GA. Specifically, numerous luminous dots emerged upon conducting a comparative analysis of SEM micrographs captured from the same location of the PLA/PBSA/LDH-GA_4 composite, utilizing both the secondary and the backscattered electron detectors. These dots were readily recognizable as LDH nanoparticles at the interface between the two polymer phases of PLA and PBSA (Figure 8). This outcome aligns with previous observations showcasing enhanced compatibility between the polymer phases, a phenomenon attributed to the role of LDH particles as effective compatibilizing agents [47].

#### 3.2.2. TGA Analysis

TGA analysis of the PLA/PBSA blend showed a two-step weight loss process, the first between 300 and 400 °C due to thermal degradation of PLA [63] with the T_max_ degradation rate at 365 °C and a second step between 350 and 450 °C due to PBSA degradation [64] with T_max_ at 397 °C. TGA thermograms of composites (Figure S4 and Table 1) showed that the T_onset_ of composites with LDH-FA and LDH-GA was at a lower temperature than PLA/PBSA, and this effect was more evident as the nanofiller content increased. For the same composites, also the T_max_ of PLA fraction was lower than that of PLA/PBSA. Interestingly, the thermal properties of the PBSA fraction seemed not influenced by the presence of the filler. Furthermore, when LDH-RA was used as a filler, both the T_onset_ and T_max_ of PLA did not change compared with that of the PLA/PBSA blend.

The literature has discussed that a decrease in PLA molecular weight can reduce both its T_onset_ and T_max_ [65,66]. The observed decrease in T_max_ for the PLA phase in the nanocomposites with LDH-FA and LDH-GA is likely due to the degradation during the mixing process at 180 °C. In fact, it has been suggested that, during the preparation of the composites containing LDH at high temperatures, the basic magnesium and aluminum hydroxides present in the lamellae of LDH, along with the adsorbed and intercalated water in the interlayer, can degrade PLA by hydrolysis [47]. Probably, composites containing LDH-FA and LDH-GA undergo such processes even if, in the case of composites with LDH-RA, the thermal properties of PLA did not change. This observation suggests that LDH-RA, with its antioxidant properties, potentially protects PLA from degradation and that PLA degradation probably did not occur only by hydrolysis but also by thermal oxidation. Nevertheless, it is plausible that the variation in the morphology of the nanocomposites, in combination with the differing degrees of interaction between the polymer matrix and the various LDHs, contributes to this effect.

#### 3.2.3. SEC Analysis

To study in detail the differences between the thermal properties of PLA/PBSA blend and composites, we analyzed by SEC both PLA and PBSA phases obtained by a separation process from the blend and the composites. The comparison of the elution curves of pure PLA e PBSA with that of PLA and PBSA separated from the blend clearly shows that the separation is possible (Figure S5).

Elution curves (Figure S6) and SEC data (Table 2) indicated that both the M_n_ and M_w_ of PLA separated from PLA/PBSA/LDH-RA_4 were similar to those of PLA separated from the blend. Instead, the M_n_ and M_w_ of PLA separated from the nanocomposites containing LDH-FA and LDH-GA were lower than those of PLA extracted from the blend. These data confirmed that the molecular weight of the PLA decreased during the preparation of the samples in the melt when LDH-FA and LDH-GA were mixed with PLA and PBSA, thus supporting the hypothesis formulated to explain the TGA results. Instead, during the preparation of the composites containing LDH-RA, the PLA molecular weight did not change. As previously discussed, the degradation of PLA by LDH during melt mixing can depend on the evaporation of adsorbed or intercalated water of LDH, which is presumed to have occurred during the mixing at 180 °C in the mechanical mixer. However, an oxidation degradation of the polymer chain can also be considered. Probably, in the case of the composite containing LDH-RA, the oxidation degradation mechanism was controlled by RA. However, the result of the composite containing LDH-FA was unexpected since FA has antioxidant activity as RA, but we observed a decrease in the molecular weight of PLA. Interestingly, the molecular weight of the PBSA phase did not change during the preparation of the composites by melt blending (Table 2), in agreement with Georgousopoulou et al. [67] who did not observe significant chain scission in PBS upon melt processing. Additionally, this outcome might be due to the preferential confinement of LDH within the PLA phase rather than in PBSA. However, further investigation is necessary to delve deeper into this aspect.

#### 3.2.4. Antioxidant Properties

We conducted a test to measure the antioxidant properties of modified LDH, specifically LDH-RA and LDH-FA (GA-H has no antioxidant properties) as well as of composites containing 4 wt.% of these materials. We utilized the DPPH method, in accordance with a previously established procedure [47]. Additionally, we compared the results of the DPPH test with those of FA-H, RA-H, and Trolox, which is a water-soluble equivalent of vitamin E. Our findings, detailed in Table 3, demonstrate that RA-H has the highest antioxidant properties and that both FA-H and RA-H have better antioxidant capacity than Trolox. When we looked at the modified LDH, we found that LDH-FA had an EC_50_ value like that of free FA-H. However, the EC_50_ of LDH-RA is higher indicating a worse antioxidant capacity than RA-H. This difference can depend on the limited availability of RA which is tightly anchored to LDH lamellae and blocked between the LDH layers.

To test if the antioxidant property of LDH-RA is transferred to PLA/PBSA composites, a film of PLA/PBSA/LDH-RA_4 was placed in a DPPH solution. The decrease in the DPPH UV-vis signal at 517 nm was measured over time. When the UV signal of DPPH reached a steady state, the same film was suspended in a fresh solution of DPPH, and the band at 517 nm was tracked again. Data (Figure S7) shows that the film still has an antioxidant activity even after the first contact with a fresh DPPH solution. In the first case, the EC_50_ value is reached in about 3 h. In the second experiment, where the film partially lost its antioxidant capacity due to the reaction with DPPH during the first experiment, the EC_50_ value was reached in 6.5 h. These results confirmed that composite films have antioxidant activity and that it is maintained over time.

#### 3.2.5. Water Vapor Permeability

The water vapor transmission rate (WVTR) determined for PLA, PLA/PBSA blend, and all the composites (Table 4) showed that PLA has a WVTR lower than PLA/PBSA. Literature reported that PBSA has higher hydrophilicity than PLA, and, in a blend of the two polymers, it can favor the hydrolysis of PLA [68]. Accordingly, it is also possible that the presence of PBSA can fast the water penetration and reduce the WVTR of the blend compared with that of PLA. Furthermore, the immiscibility of PLA and PBSA can cause the formation of microchannels and debonding at the interface between the two phases, leading to faster water penetration [69].

The presence of modified LDH in the composites improved the water vapor barrier of the blend, and the WVTR value is lower for a higher quantity of LDH in the composites. The described results fit the role of dispersed lamellar structure in a composite that, by creating a tortuous path, decreases the WVTR [70]. In contrast to the other samples, the composite with 4 wt.% of LDH-RA showed a higher WVTR value than the composite with 1 wt.%. However, the SEM analysis of PLA/PBSA_LDH-RA_4 highlighted that the dispersion and distribution of LDH-RA in this sample are scarce and showed the presence of microchannels between the two polymer phases. This morphology likely contributed to this sample’s reduced water barrier properties.

#### 3.2.6. Migration Tests and Release Kinetics

Migration tests were conducted to assess the release capacity of bioactive compounds from films of composite materials. These tests were performed on samples containing the highest LDH load (4 wt.%), and they were compared to reference samples prepared by mixing the PLA/PBSA blend with the bioactive compound in the same proportion as the composite samples. Subsequently, the films were immersed in a hydroalcoholic extraction solution, EtOH/H_2_O (50/50 *v*/*v*), commonly employed as a food simulant for lipophilic products, as European Commission Regulation No. 10/2011 indicated [71]. This simulant was preferred over water because ethanol penetration into the PLA film facilitates the relaxation of PLA macromolecular chains, thereby promoting the release of the incorporated compounds [72]. This approach allows for a more accurate assessment of the release capacity of the produced composites.

**Figure 9:**
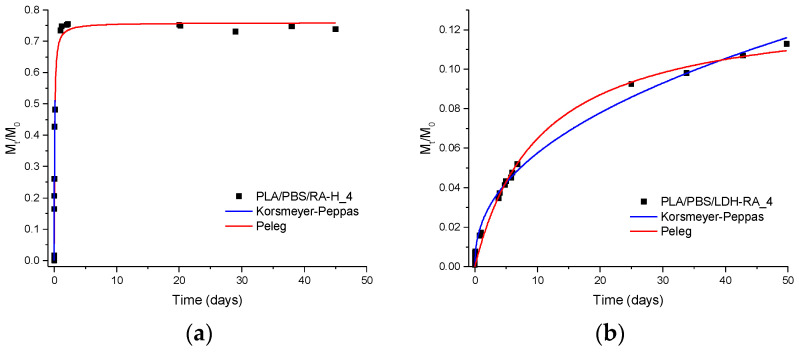
Experimental values (data points) at various contact times and fitting curves according to the Peleg model (red curve) and the Korsmeyer-Peppas model (blue curve) for the release in EtOH/H_2_O (50/50 *v*/*v*) of the active molecules present in (**a**) PLA/PBSA/RA-H_4 and (**b**) PLA/PBSA/LDH-RA_4.

The release kinetics of bioactive molecules from the films were analyzed using the Peleg model (Equation (2)) and the Korsmeyer–Peppas model (Equation (3)), both of which have been previously successfully applied to study the release of FA and cinnamic acid from thin PLA films [72]. Figure 9 presents a comparison of the experimental values of M_t_/M_0_, which represents the quantity of compound released at time t relative to the initial quantity present in the film, for the PLA/PBSA/LDH-RA_4 sample and its reference PLA/PBSA/RA-H_4. The figure also depicts the fitting curves obtained by applying both models (red curve: Peleg equation, blue curve: Korsmeyer–Peppas equation). Both selected models effectively fitted the experimental data of M_t_/M_0_. Specifically, for the PLA/PBSA/RA-H_4 sample containing free RA-H, the initial release was rapid, whereas, for the composite film PLA/PBSA/LDH-RA_4, the initial release of RA was more gradual, with a lower slope of the initial curve.

Furthermore, the value of M_t_/M_0_ at the endpoint of the experiment differed between the two samples. The maximum percentage of active molecule released from PLA/PBSA/LDH-RA_4 was approximately 12% of the ultimate value, and a steady state was not reached, unlike the reference sample containing free RA-H, which reached the plateau after only 1 h of contact with the hydroalcoholic solution. The parameters 1/k_1_ and M_∞_, obtained by fitting the data to the Peleg model, indicated that the initial release rate (1/k_1_) was three orders of magnitude higher for the reference sample containing free RA-H compared to the PLA/PBSA/LDH-RA_4 composite (Table 5). This difference in behavior can be attributed to the immobilization of RA between the LDH lamellae and the ionically bonded interaction between RA and the lamellae, resulting in a slower and controlled release of the active molecule into the extraction solution.

Regarding the parameters derived from the Korsmeyer–Peppas model, it is noteworthy that the “*k*” value exhibited a significant decrease in the LDH-RA-containing sample compared to the reference sample prepared with RA-H. This result confirmed a notably slower release of the active molecule into the extracting solution when integrated within the “host-guest” system. In contrast, the “*n*” parameter was similar for both the reference and the composite, indicating a consistent diffusion mechanism for releasing the active molecule from the PLA/PBSA matrix. Notably, the “*n*” value was close to 0.5, suggesting that the release of RA-H, whether in its acidic form or as an anion, followed a Fickian diffusion mechanism.

**Figure 10:**
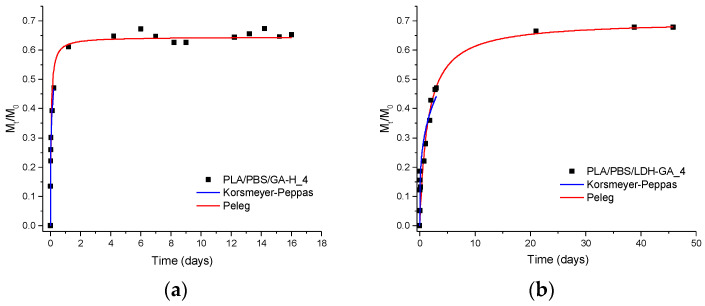
Experimental values (data points) at various contact times and fitting curves according to the Peleg model (red curve) and the Korsmeyer–Peppas model (blue curve) for the release in EtOH/H_2_O (50/50 *v*/*v*) of the active molecules present in (**a**) PLA/PBSA/GA-H_4 and (**b**) PLA/PBSA/LDH-GA_4.

Figure 10 displays both experimental data points and fitting data for the PLA/PBSA/LDH-GA_4 composite and its reference counterpart, PLA/PBSA/GA-H_4. In the case of PLA/PBSA/GA-H_4, GA-H exhibits a rapid initial release from the polymer matrix, as indicated by the sharply sloped initial curve. However, in the PLA/PBSA/LDH-GA_4 composite, the migration curve for GA follows a pattern resembling that of the reference sample, albeit with a noticeable decrease in the initial slope. In both cases, approximately 65–70% of the active molecule is released into the extraction solution upon reaching equilibrium. This substantial release is further supported by the M_∞_ values, which exceed 60% of the maximum film release capacity for both PLA/PBSA/GA-H_4 and PLA/PBSA/LDH-GA_4 samples.

The results obtained from PLA/PBSA/GA-H_4 and PLA/PBSA/LDH-GA_4 reveal significant distinctions compared to PLA/PBSA/RA-H_4 and PLA/PBSA/LDH-RA_4. This divergence can be attributed to variations in the interaction mechanisms between the constituent elements. In the LDH-RA composite, RA is intercalated into the LDH structure, forming ionic bonds. Conversely, in the LDH-GA system, GA is adsorbed onto the surface of the LDH, resulting in reduced retention within the composite and, consequently, enhanced mobility. Moreover, the adsorption-based configuration of LDH-GA may contribute to a more balanced release rate between free GA-H and GA within the LDH-GA composite. Indeed, the 1/k_1_ values for these two samples display a one-order-of-magnitude difference, in contrast to the three-order-of-magnitude difference observed in the case of RA-H and LDH-RA-based samples (see Table 5). Furthermore, the Korsmeyer–Peppas model reveals a significantly lower “*k*” value for the film containing LDH-GA compared to that containing GA-H. This outcome suggests that the lamellar system effectively delays the release of GA, thereby achieving controlled migration kinetics. In particular, the “*n*” parameter, which is less than 0.5, indicates an almost Fickian diffusion mechanism governing the release of the active molecule into the extraction solution.

The migration study was also extended to the PLA/PBSA/LDH-FA_4 composite and its respective reference, the PLA/PBSA/FA-H_4 blend. A comparison of the UV-vis spectra of the extracted solutions from the PLA/PBSA/FA-H_4 and PLA/PBSA/LDH-FA_4 samples revealed absorbance peaks at 316 nm for PLA/PBSA/FA-H (consistent with the presence of FA-H) and at 264 nm for the PLA/PBSA/LDH-FA_4 composite (Figure S8).

Since the UV-vis spectrum of the FA anion present between the layers of LDH-FA shows an absorption peak at 311 nm, it is evident that the species migrating from the PLA/PBSA/LDH-FA_4 film differs from FA. As reported in the literature, FA-H begins to degrade through decarboxylation at temperatures ranging from 150 to 200 °C, leading primarily to the formation of 4-vinyl guaiacol (4-VG), which absorbs at 260 nm as the main product, and guaiacol, resulting from the susceptibility of the vinyl double bond to oxidation [73]. In our study, since the PLA/PBSA/LDH-FA_4 sample was prepared by melt mixing at 180 °C, it is possible that localized overheating occurred due to shear stresses, even though the anion was intercalated between the layers and presumably protected from direct heat. These overheating events may have increased the temperature locally compared to the set temperature, thereby promoting the degradation of the active molecule. Indeed, as confirmation of the probable degradation of the FA anion immobilized in LDH-FA, the XRD diffractogram of the PLA/PBSA/LDH-FA_4 composite (Figure 5a) showed a weak diffraction signal at around 6°, shifted to higher angles compared to that of LDH-FA higher angles compared to LDH-FA (around 6°). This evidence suggests a contraction of the lamellar space, consistent with the formation of 4-VG.

**Figure 11:**
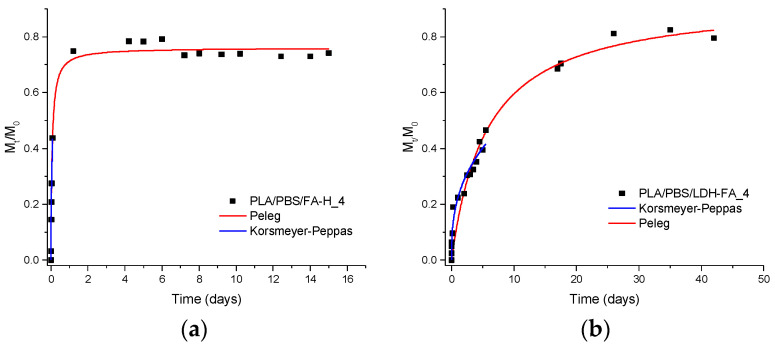
Experimental values (data points) at various contact times and fitting curves according to the Peleg model (red curve) and the Korsmeyer–Peppas model (blue curve) for the release in EtOH/H_2_O (50/50 *v*/*v*) of the active molecules present in (**a**) PLA/PBSA/FA-H_4 and (**b**) PLA/PBSA/LDH-FA_4.

We compared the migration rate in the hydroalcoholic solution of FA-H from PLA/PBSA/FA-H_4 with that of the degraded product from PLA/PBSA/LDH-FA_4, which we assumed to be 4-VG. The release of FA-H from the reference sample is rapid (Figure 11a) and reaches a steady state quickly, releasing approximately 70% of the molecule. The fitting curves for the PLA/PBSA/LDH-FA_4 sample (Figure 11b) show controlled migration but at a slower rate compared to free FA-H. Achieving a steady state takes more time compared to the reference sample. This behavior differs from previous cases because 4-VG, presumably formed, is intercalated but not ionically bound to the lamellae.

In summary, the migration process of 4-VG from PLA/PBSA/LDH-FA_4 differs from that of RA from PLA/PBSA/LDH-RA_4, as RA is intercalated and ionically bound to the lamellae, and it also differs from that of GA from PLA/PBSA/LDH-GA_4, where GA is adsorbed.

### 3.3. PLA/PBSA/LDH Characterization via Micro-Compounder

To optimize the dispersion of modified LDH in the blend and to show the possible scale-up of the nanocomposite preparation, a twin-screw micro-compounder was used to mix 1 wt.% of LDH-RA and PLA/PBSA. Besides granting a more homogeneous mixing of two immiscible polymers, extrusion processes also gave a better filler dispersion in the blend. Furthermore, it gave faster final product production. The HAAKE MiniLab with two different screw speeds has been used to prepare the samples; indeed, it is designed for compounding polymer material and online testing of rheological properties by measuring the steady-state torque of polymer melts. In all sample preparations, a slightly decreasing trend of the torque as a function of time was observed, suggesting the occurrence of some chain scission in the polymer matrix. In this type of mixer, the torque increases with the screw speed imposed by the motor power, so the torque recorded at 150 rpm was higher than one recorded at 100 rpm. The final torque was lower when modified LDH was present (Figure S9), and the difference in torque between the filled and not filled sample was higher at 150 rpm (27.3 N·cm) than at 100 rpm (12.0 N·cm), suggesting a higher degradation of polymer chains at the higher rate.

**Figure 12:**
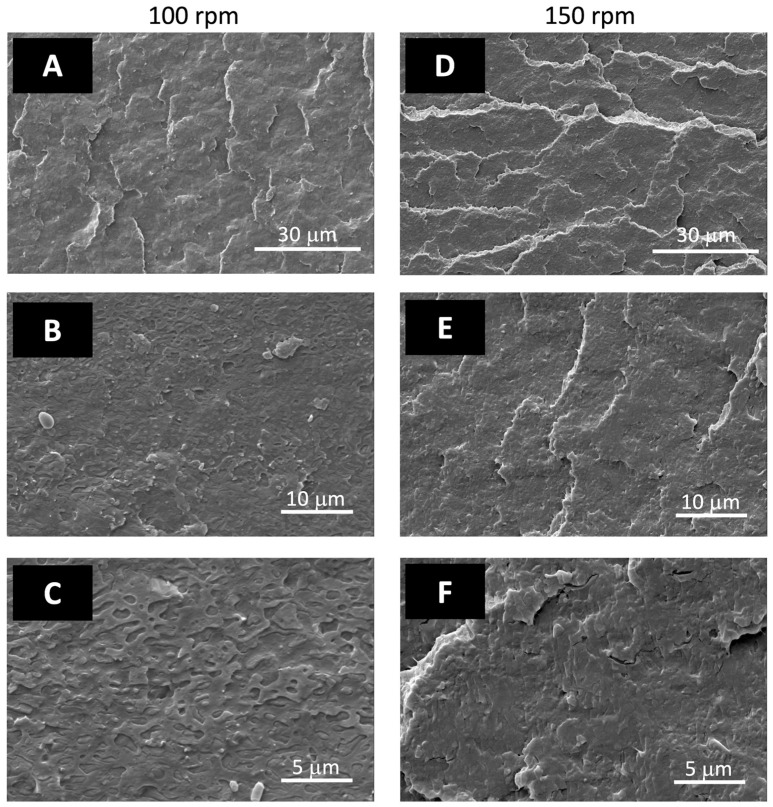
SEM micrographs of PLA/PBSA blend obtained at 100 rpm (**A**–**C**) and 150 rpm (**D**–**F**) at different magnifications.

The morphology characterization, carried out by SEM onto the cryogenic fractured surfaces of injection molded specimens, showed that the phase distribution of the PLA/PBSA 60/40 blend is typical of a co-continuous system and that a phase compatibility slight improvement was obtained by mixing PLA and PBSA at 150 rpm (compare Figure 12A–F).

**Figure 13:**
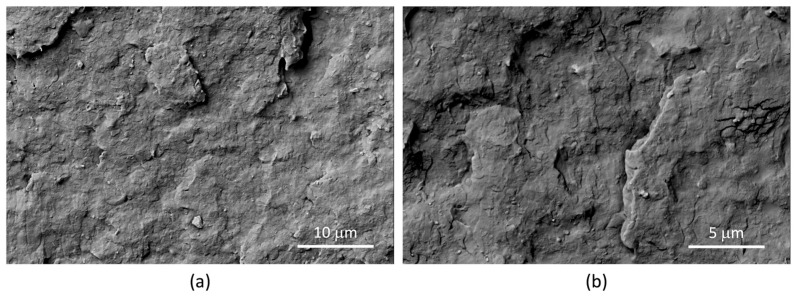
Representative backscattered electron SEM micrographs of PLA/PBSA/LDH-RA_1 prepared at 100 rpm. The micrographs were captured at various magnifications: 8000× (**a**) and 16,000× (**b**).

**Figure 14:**
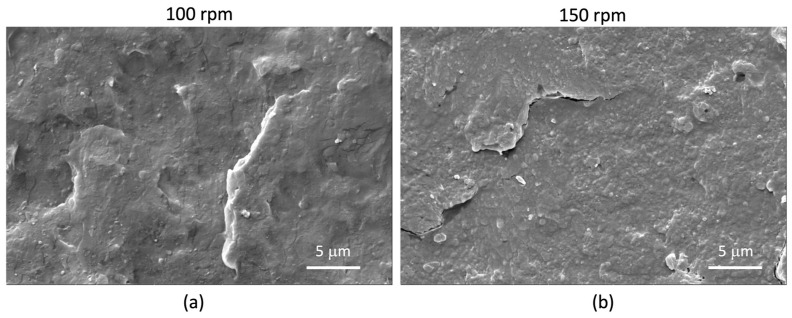
Representative SEM micrographs (ETD detector) of PLA/PBSA/LDH-RA_1 prepared at 100 rpm (**a**) and 150 rpm (**b**) at different magnifications.

The backscattered electrons micrographs of nanocomposites (Figure 13), showing a lighter color for the LDH-RA nanoparticles, allowed the identification of LDH-RA in the micrographs also collected by the EDT detector (Figure 14). The analysis of Figure 13 and Figure 14 revealed that nanocomposites showed good dispersion of LDH-RA with agglomerates smaller than 5 microns. However, despite their low concentration, some submicrometric particles homogeneously distributed can be observed. Interestingly, the nanocomposites showed increased phase compatibility, suggesting a compatibilizer role of modified LDH in the phase morphology. This effect is well evidenced by the micrograph of PLA/PBSA/LDH-RA_1 obtained at 150 rpm, where the PLA and PBSA phases are indistinguishable (Figure 14b).

The main tensile properties (Young’s modulus, yield stress, stress at break, and elongation at break) determined from the tensile stress-strain curves of PLA/PBSA prepared at 100 and 150 rpm and of the composites are reported in Table 6 and Figure S10.

An ANOVA statistical analysis of the experimental results (Table S1) reveals that the yield stress and ultimate tensile strength data are highly consistent. Indeed, the ANOVA statistical analysis of these data gives a high F-stat value and P-value lower than 0.05 that are consistent with a statistically significant difference between the means of the groups. In the case of elongation at break and Young’s modulus, the ANOVA statistical analysis gives a low F-stat value and P-value higher than 0.05, and there is not a statistically significant difference between the means of the group. Thus, the Young’s Modulus and the elongation at break were indistinguishable for all the formulations. This behavior can probably be due to the small thickness of the specimens; however, this result shows that the increase in rigidity due to the presence of the modified LDH should be considered negligible. We can also conclude that the ultimate elongation is independent of the different screw rate conditions and the addition of filler. This latter point is positive and in agreement with a good nanofiller dispersion because, generally, in the presence of agglomerates or defects, elongation at break decreases significantly. Interestingly, differences between the stress at yield values are significant, and the presence of modified LDH slightly decreases this value compared to the PLA/PBSA blend. Nevertheless, this difference is lower considering the blend and composite produced at a higher screw rate. These results confirm that increasing the screw rate can be beneficial for attaining higher compatibility between the matrix and modified LDH in agreement with papers regarding PLA composites and nanocomposites [74,75,76].

SEC analysis of PLA and PBSA phases separated from the blends and from composites prepared at 100 and 150 rpm (Table 7) shows that micro-compounder preparation caused a low decrease in the molecular weight of PLA if compared with the molecular weight of the blend prepared in the batch reactor (compare Table 2 and Table 7).

The decrease was more evident in the composites than in the blend, suggesting that the smaller quantity of LDH-RA added in these composites was insufficient to prevent molecular chain degradation. Probably, in this case, the hydrolysis of PLA polymer chains prevails over the thermal oxidation degradation mechanism. As in the case of composites and blends prepared by the batch mixer, the PBSA phase seemed not affected by degradation.

## 4. Conclusions

The FT-IR, XRD, TGA, and SEM analysis confirmed the successful intercalation of RA-H and FA-H and the immobilization, by absorption, of GA-H in LDH, all prepared by anionic exchange starting from LDH-NO_3_. Dispersion of functional LDHs in the PLA/PBSA (60/40) blend by batch reactor allowed the preparation of composites containing 1 or 4 wt.% of hybrid host-guest system. XRD analysis of the composites, especially those with 1 wt.%, evidenced an almost complete exfoliation of functional LDH, even though SEM analysis showed some sub-micrometric aggregates, especially in composites with 4 wt.% of modified LDHs. SEM analysis also showed that the blend and all composites polymer matrices have a mainly co-continuous morphology and suggested a partial compatibilization effect of modified LDHs, especially in the case of LDH-GA.

Interestingly, in composite preparation, contrary to what was observed with other modified LDHs, LDH-RA protected the PLA phase from degradation, suggesting that PLA degradation also had a thermal oxidation pathway that RA, acting as an antioxidant, can partially counteract. Indeed, we demonstrated that films of the composite with 4 wt.% of LDH-RA have antioxidant properties when soaked in a DPPH methanol solution, confirming that the LDH-RA feature can be transferred to the polymer matrix. Moreover, films of composites showed increased water barrier properties compared with the blend, and the WVTR generally increased with the amount of modified LDH.

Migration studies that were carried out on samples containing 4 wt.% of modified LDH or free organic acids, and by fitting the experimental data with the Peleg and Korsmeyer–Peppas models, showed that the free organic acids mixed with the PLA/PBSA blend migrated faster and in a larger quantity than organic carboxylates immobilized in the modified LDHs and dispersed in the blend. Thus, the immobilization of bioactive compounds in LDH slowed their migration and guaranteed a prolonged composite activity. Furthermore, the analysis of Peleg’s and Korsmeyer–Peppas’s parameters demonstrated that the migration kinetics of active molecules depends on the immobilization mode in modified LDH. Indeed, RA, which was intercalated in LDH and ionically interacting with the lamellae, was slowly released from the composites. On the other hand, GA, which was only adsorbed on the surface of LDH lamellae, was released in the fastest way. The migration of FA deserves a different comment; indeed, we observed that ferulate anion, initially present in LDH-FA, degraded, probably to 4-VG, by a decarboxylation mechanism induced by high temperature during the mixing with the PLA/PBSA blend. Interestingly, 4-VG was intercalated but not bonded to LDH, not being an anion, and its migration ability was between the immobilized RA and adsorbed GA.

Finally, we demonstrated the possibility of scaling up the production of composites containing LDH-RA (1 wt.%) using a micro-compounder and two different rotor speeds. Results of SEM analysis evidenced that the higher rotor speed allowed for better dispersion of LDH-RA in the matrix and the obtainment of a co-continuous morphology of the polymer phases. Mechanical tests on these samples confirmed the excellent dispersion of LDH-RA in the composites. Overall, these studies, exploiting nanotechnology concepts, can promote the use of biobased, biodegradable, and/or compostable and recyclable materials outperforming fossil options in smart applications, where a controlled and safe release of beneficial molecules is requested.

## Figures and Tables

**Table 1:** T_onset_ and T_max_ of PLA/PBSA and composites.

Sample	T_onset_ ^a^ (°C)	T_max_ (PLA) ^b^ (°C)	T_max_ (PBSA) ^b^ (°C)
PLA/PBSA	332	365	397
PLA/PBSA/LDH-FA_1	300	336	394
PLA/PBSA/LDH-FA_4	289	322	395
PLA/PBSA/LDH-RA_1	331	358	392
PLA/PBSA/LDH-RA_4	325	351	393
PLA/PBSA/LDH-GA_1	295	329	394
PLA/PBSA/LDH-GA_4	277	308	394

^a^ Starting degradation temperature determined as the intercept of tangents before and after the first degradation step. ^b^ Temperature corresponding to the maximum decomposition rate determined from DTG curves as the maximum of the peak.

**Table 2:** M_n_, M_w_, and dispersity (Ð) of PLA and PBSA separated from the blend and from nanocomposites.

Sample	M_n_ (g/mol)	M_w_ (g/mol)	Ð ^a^
PLA from PLA/PBSA	101,400	164,400	1.6
PLA from PLA/PBSA/LDH-FA_4	65,000	116,000	1.8
PLA from PLA/PBSA/LDH-RA_4	104,000	157,500	1.5
PLA from PLA/PBSA/LDH-GA_4	75,000	120,000	1.6
PBSA from PLA/PBSA	53,600	114,000	2.1
PBSA from PLA/PBSA/LDH-FA_4	48,000	95,000	2.0
PBSA from PLA/PBSA/LDH-RA_4	55,000	99,000	1.8
PBSA from PLA/PBSA/LDH-GA_4	53,000	90,000	1.7

^a^ Ð = M_w_/M_n_, dispersity.

**Table 3:** EC_50_ of FA-H, LDH-FA, RA-H, LDH-RA, and Trolox.

Sample	EC_50_ (µM) ^a^
FA-H	19 ± 1
LDH-FA ^b^	17 ± 1
RA-H	2.7 ± 0.1
LDH-RA ^b^	19 ± 7
Trolox	23 ± 3

^a^ Mean value of three runs ± standard deviation. ^b^ EC_50_ values were calculated considering the theoretical molar concentration of A-H available in LDH-RA (46 wt.%) and LDH-FA (37 wt.%).

**Table 4:** Water vapor transmission rate (WVTR) for PLA, PLA/PBSA blend, and composites.

Sample	WVTR (g/m^2^ × 24 h) ^a^
PLA	13.5
PLA/PBSA	19.0
PLA/PBSA/LDH-FA_1	18.5
PLA/PBSA/LDH-FA_4	17.8
PLA/PBSA/LDH-RA_1	13.4
PLA/PBSA/LDH-RA_4	24.1
PLA/PBSA/LDH-GA_1	17.0
PLA/PBSA/LDH-GA_4	16.7

^a^ WVTR values are normalized to the thickness of the film.

**Table 5:** Parameters for the Peleg and Korsmeyer–Peppas models extracted through nonlinear fitting of experimental data points for the PLA/PBS_RA-H_4, PLA/PBS_LDH-RA_4, PLA/PBS_GA-H_4, and PLA/PBS_LDH-GA_4 samples.

Sample	Peleg				Korsmeyer-Peppas		
	1/k_1_ ^a^ (mg/Day)	M_∞_ ^b^ (mg/100 mg Film)	M_∞_/M_0_ ^c^ (%)	R^2 d^	n ^e^	k ^f^ (Day^−n^)	R^2 d^
PLA/PBSA/RA-H_4	20 ± 2	1.43 ± 0.02	77 ± 1	0.99	0.47 ± 0.06	1.6 ± 0.2	0.97
PLA/PBSA/LDH-RA_4	0.023 ± 0.002	0.242 ± 0.007	13.2 ± 0.4	0.99	0.44 ± 0.01	0.19 ± 0.01	0.99
PLA/PBSA/GA-H_4	7 ± 1	0.367 ± 0.008	64 ± 1	0.96	0.26 ± 0.01	1.05 ± 0.02	0.99
PLA/PBSA/LDH-GA_4	0.28 ± 0.07	0.40 ± 0.02	70.1 ± 0.5	0.89	0.28 ± 0.04	0.48 ± 0.01	0.91

^a^ Peleg model: initial release rate of the active compound. ^b^ Peleg model: amount of active compound released at equilibrium. ^c^ Peleg model: percentage of active compound released at equilibrium. ^d^ Coefficient of determination of the model. ^e^ Korsmeyer–Peppas model: diffusional exponent indicating the transport mechanism. ^f^ Korsmeyer–Peppas model: release rate constant of the active molecule.

**Table 6:** Tensile properties for PLA/PBSA and composites containing 1 wt.% of LDH-RA prepared at 100 and 150 rpm ^a^.

Sample	Young’s Modulus (GPa)	Yield Stress (MPa)	Stress at Break (%)	Elongation at Break (%)
PLA/PBSA_100	1.8 ± 0.1	38.1 ± 0.2	29.9 ± 0.5	240 ± 41
PLA/PBSA/LDH-RA_1_100	1.9 ± 0.2	34 ± 1	27 ± 1	206 ± 67
PLA/PBSA/150	2.0 ± 0.2	40 ± 1	29.9 ± 0.2	244 ± 41
PLA/PBSA/LDH-RA_1_150	2.1 ± 0.2	38 ± 1	27.0 ± 0.3	227 ± 48

^a^ Data were means of triplicate measurements ± Standard Deviation (n = 3).

**Table 7:** SEC data of PLA and PBSA phases separated from PLA/PBSA blend and composites prepared by micro-compounder at 100 and 150 rpm.

Sample	Mn (g/mol)	Mw (g/mol)	Ð ^1^
PLA from PLA/PBSA_100	76,500	149,000	1.9
PLA from PLA/PBSA_150	78,000	155,000	2.0
PLA from PLA/PBSA/LDH-RA_1_100	66,300	145,500	2.2
PLA from PLA/PBSA/LDH-RA_1_150	62,700	138,000	2.2
PBSA from PLA/PBSA_100	59,000	127,000	2.2
PBSA from PLA/PBSA_150	53,700	128,000	2.4
PBSA from PLA/PBSA/LDH-RA_1_100	57,000	117,000	2.1
PBSA from PLA/PBSA/LDH-RA_1_150	57,000	118,000	2.1

^1^ Ð = Mw/Mn, dispersity.

## Data Availability

Data is contained within the article or Supplementary Material.

## Supplementary Materials

The following supporting information can be downloaded at: https://www.mdpi.com/article/10.3390/jfb14110549/s1, Figure S1: FT-IR spectra of LDH-NO_3_, LDH-FA, LDH-RA, and LDH-GA; Figure S2: Torque curves versus time recorded during batch mixing for the preparation of PLA/PBSA blend and composites; Figure S3: SEM micrograph of PLA/PBSA blend; Figure S4: TGA and fist derivative (DTG) of PLA/PBSA and composites with 1 wt.% and 4 wt.% (a) LDH-FA, (b) LDH-RA, and (c) LDH-GA; Figure S5: Comparison between the elution curves of PLA/PBSA, pure PLA, pure PBSA, and PLA and PBSA separated from PLA/PBSA blend. Curves are normalized. Figure S6: Comparison between the elution curves of (A) pure PLA and PLA separated from PLA/PBSA blend and from composites containing 4 wt.% of modified LDH. (B) pure PBSA and PBSA separated from PLA/PBSA blend and from composites containing 4 wt.% of modified LDH. Curves are normalized; Figure S7: I% as a function of contact time of PLA/PBSA/LDH-RA_4 with DPPH; Figure S8: Comparison of UV-vis spectra between the extracting solution EtOH/H_2_O (50/50 *v*/*v*) in contact with films of PLA/PBSA/FA-H_4 and PLA/PBSA/LDH-FA_4. Figure S9: Torque behavior as a function of time for the preparation of PLA/PBSA blend and composites containing 1 wt.% of LDH-RA. Figure S10: Mechanical tests results. Table S1: ANOVA statistical results.
