# Supplementary material for: New Functional Bionanocomposites by Combining Hybrid Host-Guest Systems with a Fully Biobased Poly(lactic acid)/Poly(butylene succinate-co-adipate) (PLA/PBSA) Binary Blend
Source: J Funct Biomater. 2023 Nov 15;14(11):549. doi: 10.3390/jfb14110549 (PMC10672472; doi:10.3390/jfb14110549)
Supplement: Supplementary file 1 [file jfb-14-00549-s001.zip › jfb-2664946-supplementary.pdf]

Journal of Functional Biomaterials

Supplementary Materials

New Functional Bionanocomposites by Combining Hybrid Host-Guest Systems with a Fully Biobased Poly(lactic Acid)/Poly(butylene Succinate-Co-Adipate) (PLA/PBSA) Binary Blend

Francesca Cicogna [1,*], Elisa Passaglia [1], Alice Telleschi [1], Werner Oberhauser [2], Maria-Beatrice Coltelli [3], Luca Panariello [3], Vito Gigante [3] and Serena Coiai [1,*]

1 National Research Council-Institute for the Chemistry of OrganoMetallic Compounds (CNR-ICCOM), SS Pisa, Via Moruzzi 1, 56124 Pisa, Italy; elisa.passaglia@pi.iccom.cnr.it (E.P.); alitelleschi@gmail.com (A.T.)
2 National Research Council-Institute for the Chemistry of OrganoMetallic Compounds (CNR-ICCOM), Via Madonna del Piano 10, 50019 Sesto Fiorentino, Italy; woberhauser@iccom.cnr.it (W.O.)
3 Department of Civil and Industrial Engineering, University of Pisa, Largo L. Lazzarino 1, 56122 Pisa, Italy; maria.beatrice.coltelli@unipi.it (M.-B.C.); luca.panariello@ing.unipi.it (L.P.); vito.gigante@dici.unipi.it (V.G.)
* Correspondence: francesca.cicogna@pi.iccom.cnr.it (F.C.); serena.coiai@pi.iccom.cnr.it (S.C.); Tel.: +39-050-3153393 (F.C.); +39-050-3152556 (S.C.)

Citation: Cicogna, F.; Passaglia, E.; Telleschi, A.; Oberhauser, W.; Coltelli, M.-B.; Panariello, L.; Gigante, V.; Coiai, S. New Functional Bionanocomposites by Combining Hybrid Host-Guest Systems with a Fully Biobased Poly(lactic acid)/Poly(butylene succinate-co-adipate) (PLA/PBSA) Binary Blend. *J. Funct. Biomater.* **2023**, *14*, 549. https://doi.org/10.3390/jfb14110549

Academic Editor: Piotr Kurcok

Received: 29 September 2023
Revised: 25 October 2023
Accepted: 7 November 2023
Published: 15 November 2023

Copyright: © 2023 by the authors. Submitted for possible open access publication under the terms and conditions of the Creative Commons Attribution (CC BY) license (https://creativecommons.org/licenses/by/4.0/).

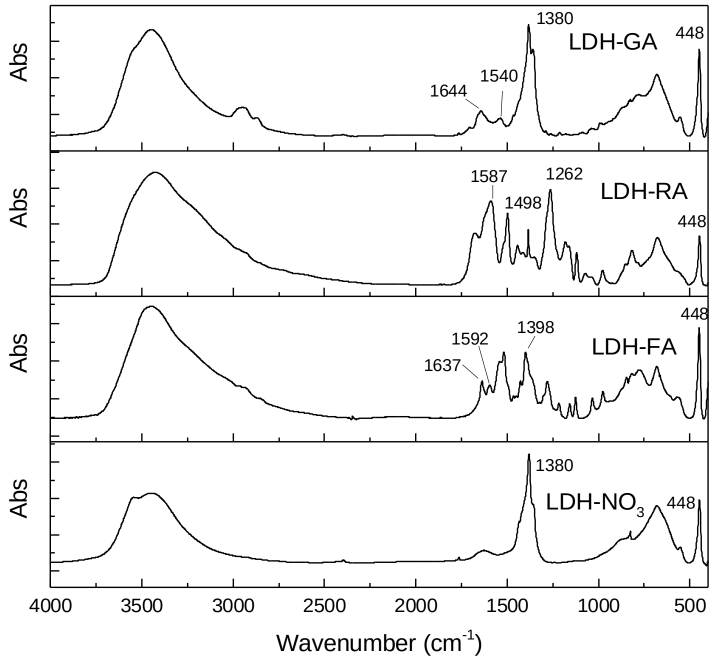

Figure S1. FT-IR spectra of LDH-NO_3, LDH-FA, LDH-RA, and LDH-GA.

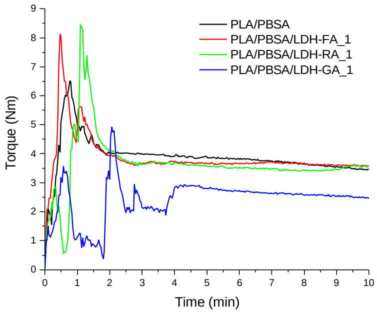

Figure S2. Torque curves versus time recorded during batch mixing for the preparation of PLA/PBSA blend and composites.

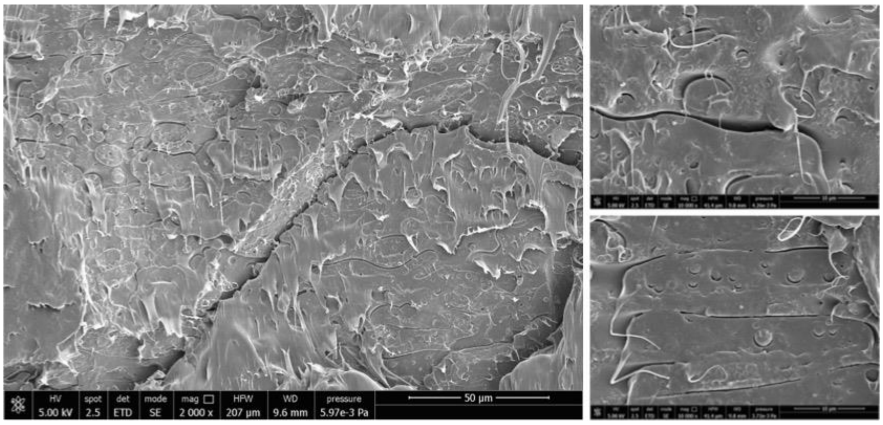

Figure S3. SEM micrograph of PLA/PBSA blend.

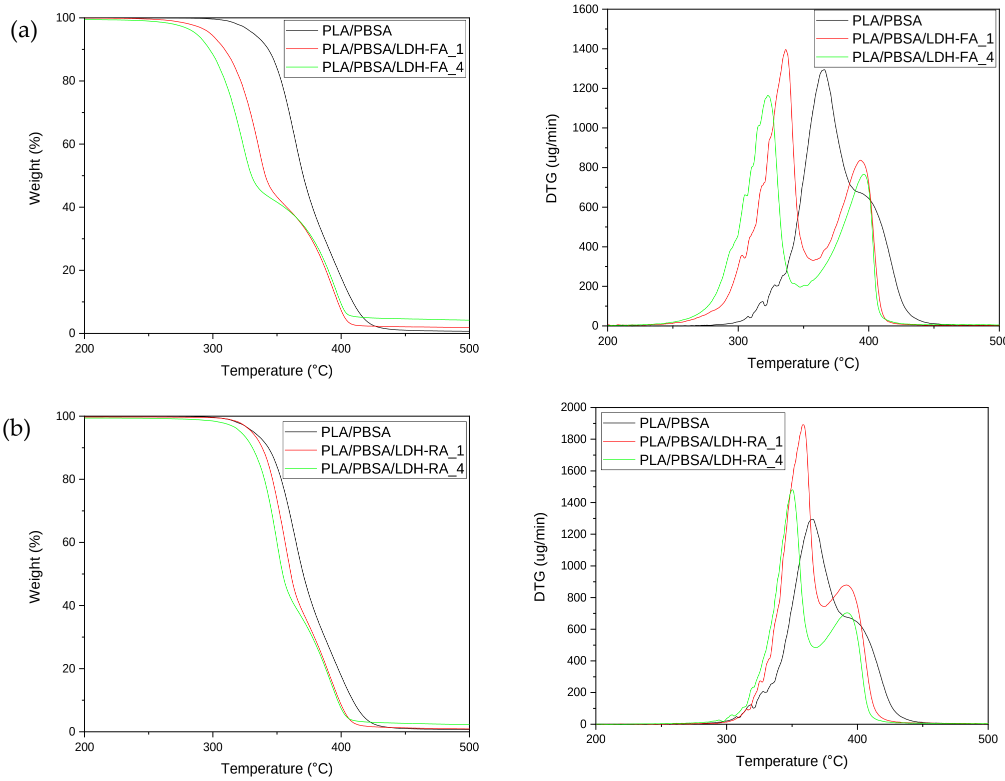

(c)

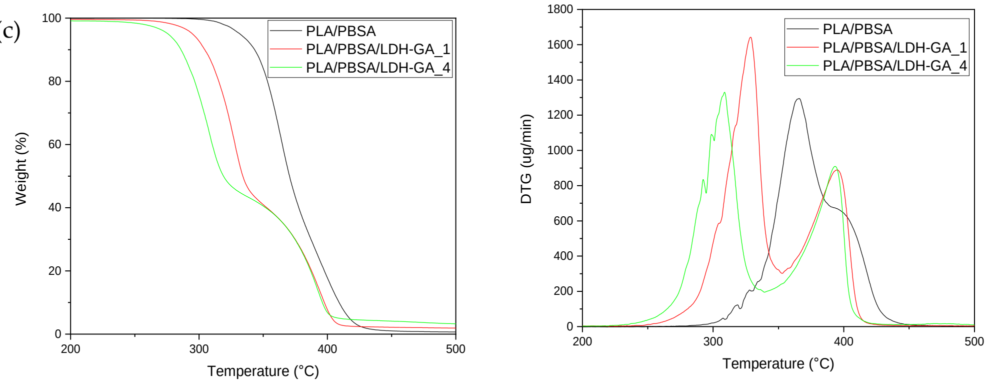

Figure S4. TGA and fist derivative (DTG) of PLA/PBSA and composites with 1 wt.% and 4 wt.% (**a**) LDH-FA, (**b**) LDH-RA, and (**c**) LDH-GA.

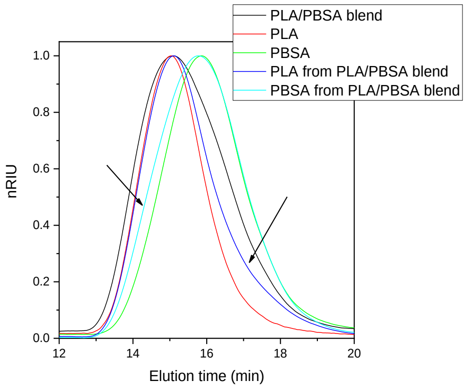

Figure S5. Comparison between the elution curves of PLA/PBSA, pure PLA, pure PBSA, and PLA and PBSA separated from PLA/PBSA blend. Curves are normalized.

(a)

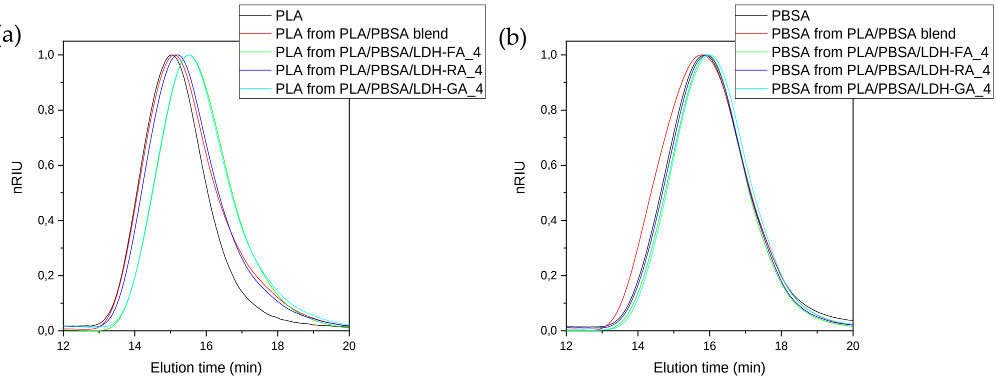

Figure S6. Comparison between the elution curves of (**a**) pure PLA and PLA separated from PLA/PBSA blend and from composites containing 4 wt.% of modified LDH. (**b**) pure PBSA and PBSA separated from PLA/PBSA blend and from composites containing 4wt.% of modified LDH. Curves are normalized.

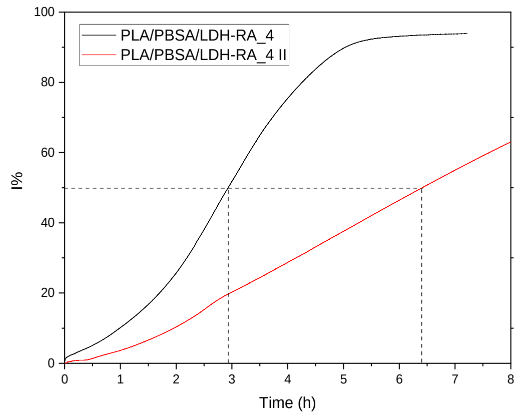

Figure S7. I% as a function of contact time of PLA/PBSA/LDH-RA_4 with DPPH.

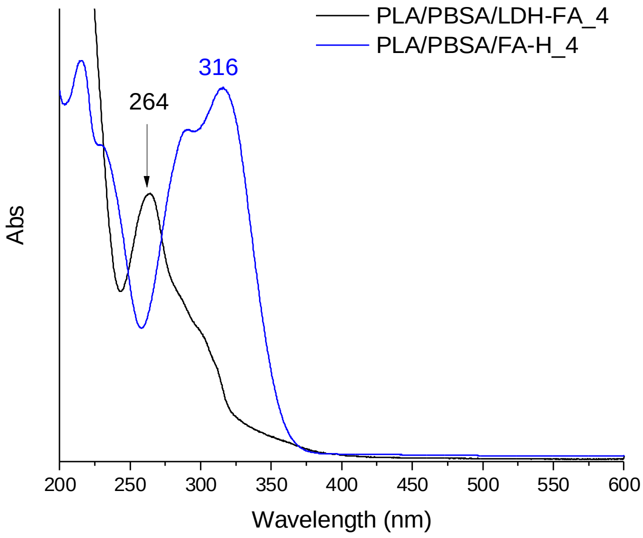

Figure S8. Comparison of UV-vis spectra between the extracting solution EtOH/H_2O (50/50 v/v) in contact with films of PLA/PBSA/FA-H_4 and PLA/PBSA/LDH-FA_4.

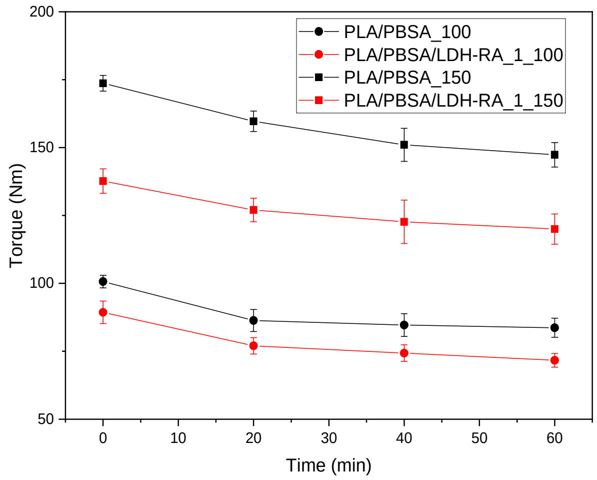

Figure S9. Torque behavior as a function of time for the preparation of PLA/PBSA blend and composites containing 1 wt.% of LDH-RA.

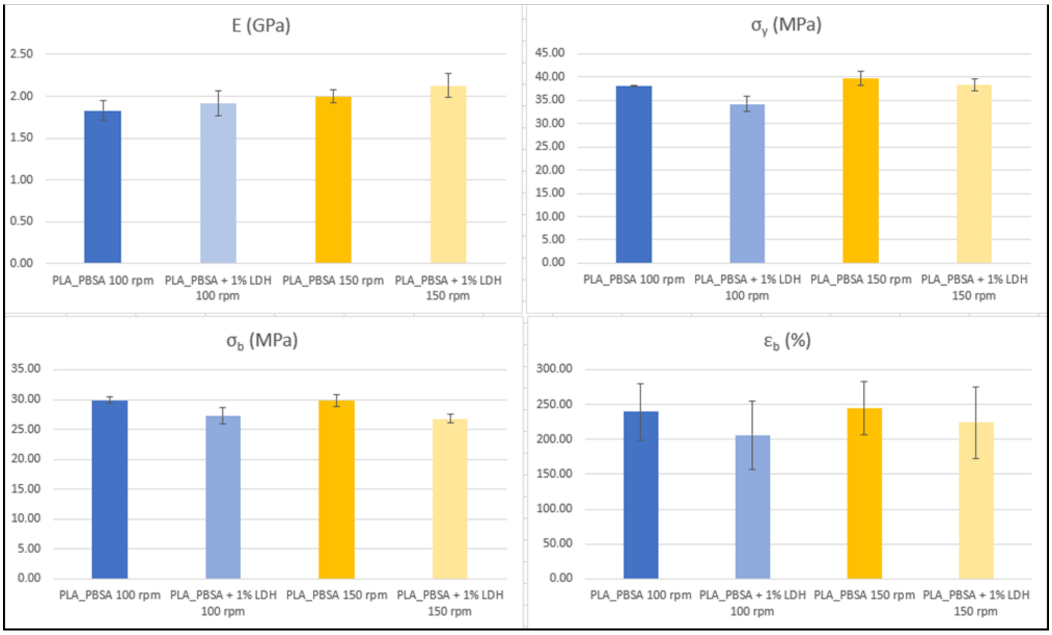

Figure S10. Mechanical tests results.

Table S1. ANOVA statistical results.

**Source**	**Degrees of Freedom (DF)**	**Sum of Squares (SS)**	**Mean Square (MS)**	**F-Stat**	**P-Value**
Elastic Modulus	3	0.0868	0.0289	1.5255	0.2809
Yield Strength	3	48.3671	16.1224	9.5028	0.0052
Stress at break	3	23.1212	7.7071	12.6502	0.0021
Elongation at break	3	2594.352	864.7839	0.3418	0.7961
